# The Relationship Between Regulatory Frameworks for Protein Content Claims for Plant Protein Foods and the Nutrient Intakes of Canadian Adults

**DOI:** 10.3390/nu17182987

**Published:** 2025-09-18

**Authors:** Songhee Back, Christopher P. F. Marinangeli, Antonio Rossi, Lamar Elfaki, Mavra Ahmed, Victoria Chen, Shuting Yang, Andreea Zurbau, Alison M. Duncan, Mary R. L’Abbe, Cyril W. C. Kendall, John L. Sievenpiper, Laura Chiavaroli

**Affiliations:** 1 Department of Nutritional Sciences, Temerty Faculty of Medicine, University of Toronto, Toronto, ON M5S 1A8, Canada; songhee.back@mail.utoronto.ca (S.B.); antonio.rossi@mail.utoronto.ca (A.R.); mavz.ahmed@utoronto.ca (M.A.); tori.chen@mail.utoronto.ca (V.C.); queenie.yang@mail.utoronto.ca (S.Y.); andreea.zurbau@mail.utoronto.ca (A.Z.); mary.labbe@utoronto.ca (M.R.L.); cyril.kendall@utoronto.ca (C.W.C.K.); john.sievenpiper@utoronto.ca (J.L.S.); 2 Toronto 3D Knowledge Synthesis and Clinical Trials Unit and Clinical Nutrition and Risk Factor Modification Centre, St. Michael’s Hospital, Toronto, ON M5B 1W8, Canada; 3 Center for Regulatory Research and Innovation, Protein Industries Canada, Regina, SK S4P 1Y1, Canada; 4 Faculty of Arts and Science, University of Toronto, Toronto, ON M5S 1A1, Canada; lamar.elfaki@mail.utoronto.ca; 5 Joannah & Brian Lawson Centre for Child Nutrition, Temerty Faculty of Medicine, University of Toronto, Toronto, ON M5S 1A1, Canada; 6 INQUIS Clinical Research Ltd. (Formerly GI Labs), Toronto, ON M5C 2N8, Canada; 7 Department of Human Health and Nutritional Sciences, University of Guelph, Guelph, ON N1G 2W1, Canada; amduncan@uoguelph.ca; 8 College of Pharmacy and Nutrition, University of Saskatchewan, Saskatoon, SK S7N 5A2, Canada; 9 Division of Endocrinology and Metabolism, Department of Medicine, St. Michael’s Hospital, Toronto, ON M5B 1W8, Canada; 10 Department of Medicine, Temerty Faculty of Medicine, University of Toronto, Toronto, ON M5S 1A1, Canada; 11 Li Ka Shing Knowledge Institute, St. Michael’s Hospital, Toronto, ON M5B 1W8, Canada

**Keywords:** protein, nutrition content claim, protein content claim, food labelling, protein quality, plant-based protein, protein efficiency ratio, protein digestibility corrected amino acid score (PDCAAS), digestible corrected amino acid score (DIAAS), Canadian Community Health Survey (CCHS)

## Abstract

**Background:** The inability to assign a protein content claim (PCC) to plant foods may impede efforts from Canada’s Food Guide to increase consumption of plant protein. A systematic application of PCC frameworks from other regions to Canadian nutrition surveillance data would be useful to model potential effects of PCC regulations on the nutrient intake, protein quality, and corrected protein intake of diets. **Methods:** Plant food groups that qualified for a PCC within the Canadian Nutrient File according to regulations from Canada, the United States (US), Australia and New Zealand (ANZ), and the European Union (EU) were identified. Adults (≥19 years) (n = 11,817) from The Canadian Community Health Survey (2015) who consumed ≥1 plant food qualifying for a PCC in each region were allocated to the corresponding PCC group. The effects of Canadian PCC regulations on the protein quantity, quality (Digestible Indispensable Amino Acid Score, DIAAS), and nutrient intakes of Canadian diets in adults were compared to PCC groups from other regions. **Results:** Substantially more individuals were consumers of plant-based protein foods, using the ANZ and the EU PCC regulations, compared to the Canadian and US PCC groups. There were no differences in uncorrected protein intake across PCC groups. All DIAAS values were >0.94, and corrected protein intakes were >74–89 g/day or 16%E across PCC groups. Non-consumers of plant foods eligible for a PCC had corrected protein intakes that ranged between 68 and 78 g/d or 17%E. Generally, consumers of plant foods eligible for a PCC in the US, ANZ, and EU, or both Canada and the US/ANZ/EU, had higher intakes of positive nutrients, such as fibre, calcium, iron, magnesium, and zinc (*p* < 0.05) and lower saturated fat. **Conclusions:** Less restrictive regulatory frameworks for PCC used in ANZ and the EU did not substantially affect protein intake or the protein quality of Canadian diets in adults. These results suggest that more inclusive regulatory frameworks for protein PCCs could support increased intake of food sources of plant proteins in alignment with Canada’s Food Guide.

## 1. Introduction

The most recent iteration of Canada’s Food Guide (CFG) encourages Canadians to consume “plant-based protein more often” to enhance the nutrient density, increase dietary fibre, displace saturated fat content, and lower the environmental impacts of diets [1]. Similar recommendations appear in dietary guidelines from other jurisdictions [2,3,4]. Shifting dietary patterns to include more plant protein foods offers a significant opportunity to address public health challenges associated with cardiovascular disease (CVD) and diabetes [5,6,7,8].

Food and beverage labelling can influence consumers’ dietary choices and is a key strategy to support the consumption of nutrients, including those in alignment with Canada’s Healthy Eating Strategy and CFG [9]. Nutrient content claims, such as protein content claims (PCC), help consumers identify foods that are a ‘source of protein’, including plant-based options. PCCs are generally quantified by the amount of protein in a food. However, Canada and the US are the only two high-income jurisdictions that use protein quality (PQ) in conjunction with protein quantity as support for PCC for non-infant and non-medical foods.

Until recently, the protein efficiency ratio (PER) was the only regulated method for determining the PQ of foods in Canada to establish a protein rating to support a PCC [10]. The PER method is a rat growth bioassay and presents several limitations. It does not account for amino acid requirements for maintenance, it cannot be used to theoretically calculate the PER of foods that combine various sources of protein, and the amino acid requirements of rats differ from those of humans [11,12]. Therefore, the PER has been deemed outdated/limited for evaluating protein in complex, non-infant human diets [11].

The US also uses PQ to qualify protein claims using the Protein Digestibility Corrected Amino Acid Score (PDCAAS). PDCAAS corrects the level of crude protein in a food based on the true nitrogen (N) digestibility of the protein and indispensable amino acid (IAA) requirements for 2–5-year-old children (mg/g protein) [13], coinciding with the FAO’s 1991 report [11]. Other jurisdictions, such as Australia and New Zealand (ANZ) and the European Union (EU), use absolute levels of crude protein or energy from protein to support PCCs, respectively [14,15].

With the exception of soy and a few other plant foods, the PQ of plant foods is generally lower than that of animal foods. Thus, the application of PQ to support PCCs can limit the ability for many plant foods to make “source of protein” claims on food labels in Canada and the US compared to other regions such as ANZ or the EU [16]. Given the use of PER and its aforementioned challenges, Canada’s PCC framework has been considered to be more restrictive and significantly different from those in other jurisdictions [12].

The restrictive Canadian regulations for PCCs can prevent the identification of plant foods with significant levels of protein in the retail environment by consumers, limiting adherence to CFG. The discrete application of PQ of a food at a single eating occasion also fails to consider its contribution to protein and amino acid complementarity. Various plant protein foods, such as legumes, nuts, and seeds, can be combined with cereals and animal proteins to complement differences in IAA levels between foods, where at a meal and/or over the course of a day sufficient IAA are consumed [17]. While PQ is an important measure for assessing sufficient IAA and protein intake, particularly for sole-sourced foods such as infant foods, the application of PQ in the context of PCCs can limit food choices and challenge alignment with CFG. Regulatory modernization of PCC frameworks in Canada to better support the consumption of a broader range of sources of plant protein and foster a holistic approach to dietary patterns by consumers is warranted.

The objective of this study was to model and assess PQ, protein quantity, and nutrient intakes of Canadians who consume plant protein foods eligible for a PCC under regulatory frameworks in Canada, the US, ANZ, and the EU (PCC groups) compared to non-consumers. It is hypothesized that the application of more inclusive PCC frameworks will increase the number of plant foods eligible for a PCC without compromising the protein intake or PQ of Canadian diets.

## 2. Materials and Methods

A more detailed overview of Materials and Methods is provided in Supplemental Materials.

### 2.1. Data Sources

#### 2.1.1. The 2015 Canadian Community Health Survey—Nutrition (CCHS)

The CCHS is a cross-sectional, voluntary, national health survey conducted by Statistics Canada [18]. CCHS is a representative sample of the population across 10 Canadian provinces. Individuals who were full-time members of the Canadian Forces, living in Canada’s two territories, on reserves, in Aboriginal settlements, or in institutions were excluded. A complete description of the sampling method used for the CCHS is provided elsewhere [18]. Nutrition Public Use Microdata Files (PUMF) from the CCHS were used to quantify food intakes. This is the first open-access PUMF released for CCHS. It contained all the information required for this study, such as demographics, dietary patterns (including details on what foods were eaten and quantities), health information—including self-reported physical activity, height, and weight—and health conditions [19].

A detailed overview of the CCHS is provided elsewhere [18]. Briefly, dietary data were collected using the United States Department of Agriculture’s five-step automated multi-pass method, adapted and modified for the Canadian population to conduct computer-assisted interviews [20]. Survey interviews were conducted between 2 January 2015 and 31 December 2015. Overall, 20,487 individuals completed the initial 24-h dietary recall. A random sub-sample (n = 7608) completed the second recall by phone 3–10 days after. Response rates were 61.6% and 68.6%, respectively, for the initial and second recall. For the present analysis, the first 24-h dietary recall from the 2015 CCHS-Nutrition PUMF data was used [19].

#### 2.1.2. Inclusion and Exclusion Criteria for Data Collection

**Figure 1:**
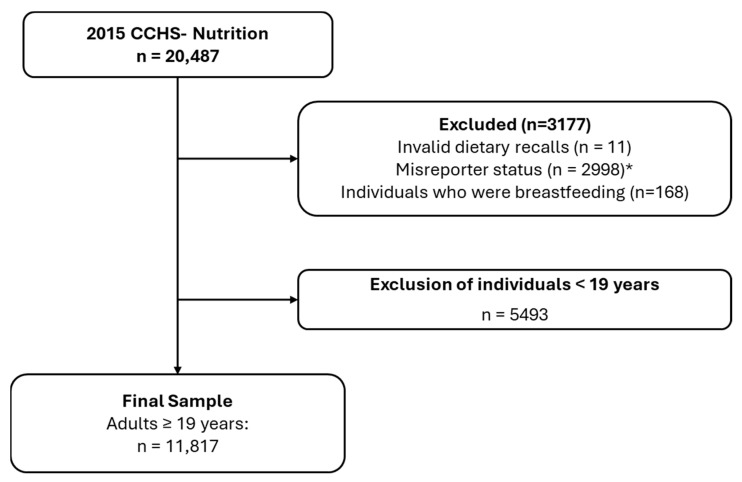
Sample selection process. * Did not have relevant information to adjust for misreporting status based on the previously published method [21,24].

Sampling criteria for this study are summarized in Figure 1. Participants with invalid dietary recalls or reporting no food or energy intake were excluded (n = 11).

As misreporting was used as a covariate in all analyses, those identified as respondents who lacked sufficient information to adjust for misreporting status were also excluded (n = 2998). Misreporting status was based on previously published methods that compared reported energy intake (EI) and total energy expenditure (TEE) (i.e., overreporting and underreporting of EI) [21]. The TEE of respondents was predicted based on the variables age, sex, height, weight, and physical activity levels using the Institute of Medicine’s equations [22]. Body Mass Index (BMI) was calculated using measured height and weight for the respondents. When not measured, a correction factor was applied to the adults with self-reported height and weight, and sex-adjusted [23]. Cut-offs to define sedentary, low active, active, and very active for physical activity levels were applied based on Health Canada’s Guidance [18]. Underreporting and overreporting were defined as the ratio of EI/TEE and used as a misreporting variable to determine the misreporting status of dietary recalls. Therefore, underreporting and overreporting were defined as the ratio of EI/TEE < 0.7 and >1.42, respectively, whereas those in between were considered plausible reporters [21]. Given that there was no TEE equation for underweight subjects, all individuals with a BMI < 18.5 kg/m^2^ (n = 377) were excluded.

All individuals < 19 years (n = 5493) and participants who were breastfeeding (n = 168) were also excluded [20,25]. Pregnant individuals were not identified in the survey. After exclusions, the final sample size was 11,817 adults (≥19 years), which was pooled data from male (n = 5670) and female (n = 6147) sex groups.

#### 2.1.3. The Canadian Nutrient File and Bureau of Nutritional Science Food Groups

For the nutritional analysis of foods, the CCHS was coded by Health Canada using the 2015 Canadian Nutrient File (CNF) and Health Canada Bureau of Nutritional Science (BNS) food groups [18,25]. The CNF is a national food and nutrient database that reflects the nutrient composition of foods in Canada. Since the completion of the CCHS, the CNF has undergone some updates. For this study, the 2018 CNF was downloaded on 23 July 2019, and included macronutrient and micronutrient composition data of 5596 foods. Each food from the CNF is classified under a BNS food group, then subclassified under a subgroup (Supplemental Table S1).

To more accurately aggregate the protein and IAA levels of plant BNS food groups, a number of changes to the BNS food group structure were made for this study (Supplemental Tables S2–S6). Additionally, five foods were reassigned to new food groups due to misclassification and better alignment to how they are consumed in dietary patterns (Supplemental Table S2, items 7–9).

In preparation for determining whether plant BNS food groups were eligible for a PCC across regions (Section 2.2), additional food exclusions were made to prevent redundancies, account for cooked vs. raw composition, and overweighting of similar foods within plant BNS food groups (Supplemental Table S7).

### 2.2. PCC Validation for BNS Food Groups for Each Region

**Figure 2:**
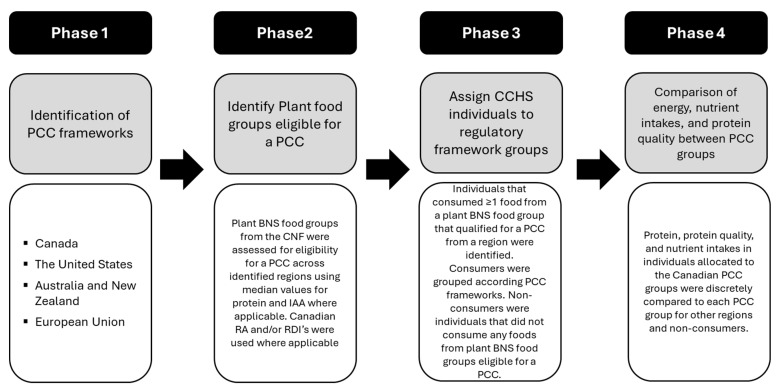
Summary of the methodology applied to evaluate the eligibility of plant BNS food groups for a PCC across regions and subsequent application to Canadian surveillance data to model the potential effects of PCC regulations on the protein, protein quality, and nutrient intakes of Canadians. CCHS, 2015 Canadian Community Health Survey—Nutrition; CNF, Canadian Nutrient File; IAA, indispensable amino acids; PCC, protein content claim; RA, reference amount; RDI, reasonable daily intake.

A stepwise summary of the methods across Section 2.2, Section 2.3 and Section 2.4 is provided in Figure 2.

#### 2.2.1. Summary of PCC Regulatory Frameworks for Canada, the US, ANZ, and the EU

Canada’s PCC regulatory framework was compared with those of the US, ANZ, and the EU. These PCC frameworks were chosen because these regions are governed by like-minded regulatory agencies [26,27] and have similar food systems compared to Canada. PCC frameworks are summarized in Table 1, where each region permits two levels of PCCs: “source” and “high source”. Detailed explanations for calculating the PCC of these four regulatory frameworks have been previously described [28] and are briefly summarized below.

Methods for assessing the PQ for eligibility for a PCC in Canada and the US are summarized in Supplemental Figures S1–S3. For Canada, the PER rat growth bioassay involves feeding growing weanling rats either the test protein or a casein (control) as the source of protein [32] (Supplemental Figure S1). The protein rating of a food is determined by multiplying the adjusted PER by the protein content in a Reasonable Daily Intake (RDI) of a specific food. The RDI is a regulated level of intake corresponding to the amount of a food typically consumed in a single day in Canada [33]. When an RDI for a food does not exist, the amount of protein in a reference amount (RA) of the food can be used [34]. The RA is a regulated serving size of a food in Canada based on historical consumption [35]. In Canada, using the PER method, a protein rating of ≥20 qualifies for a “good source”, and ≥40 qualifies for an “excellent source” of protein claim [29].

In December 2020, given the ongoing logistical challenges with the utilization of the PER, the Government of Canada provided industry with an interim policy for generating a PER from a known PDCAAS for a food to support a PCC [12,36,37,38] (Supplemental Figure S2). It is important to note that the relationship between in vivo PER determination and derivation of PER from PDCAAS has not been scientifically validated [28].

In the US, the PDCAAS of a food is calculated according to methods outlined by the FAO’s 1991 report [13] using true N digestibility coefficients and IAA (mg/g protein) of the food [11] (Supplemental Figure S3). An amino acid score (AAS) is calculated by dividing the level of each IAA (mg/g protein) in the food by the corresponding IAA (mg/g protein) requirements from the reference pattern for children 2–5 years of age as per the FAO’s 1991 report [11]. The amino acid with the lowest score is multiplied by the digestibility coefficient to give a PDCAAS. For foods with multiple sources of protein, a weighted true N digestibility coefficient can be applied [11]. The PDCAAS is multiplied by the level of protein per Reasonable Amount Customarily Consumed (RACC) of a food to provide a corrected level of protein. Similar to the RA in Canada, a RACC is a regulated serving size. A PDCAAS of 1.0 indicates that all IAA requirements (mg/g protein) are being met by the food. All PDCAAS values > 1.00 are truncated to 1.00 [11]. When the corrected level of protein is ≥10% and ≥20% of the daily value for protein (50 g/day) [13], the food can qualify for a “good” or “excellent” source of protein, respectively [31].

PCCs in ANZ and the EU are based on protein quantity or energy from protein, per serving, respectively. ANZ assesses the amount of protein in grams per serving, with ≥5 g per serving required for a “general source” and ≥10 g per serving for a “good source” of protein claim [14]. The EU measures the percentage of energy from protein where ≥12% energy per serving is a “source” and ≥20% energy per serving is a “high source” of protein [15].

#### 2.2.2. Assessment of Plant BNS Food Groups for a PCC Under Canadian, US, ANZ, and EU Regulations

Assumptions and data used to calculate the PQ and/or eligibility for a PCC for plant BNS food groups are summarized in Supplemental Table S8.

##### Eligibility of BNS Food Groups for a PCC Under Canadian Regulations

To assess the eligibility of a BNS food group for a PCC under Canadian regulations, Canada’s PER PCC regulations were used alongside the interim PDCAAS policy in place at the time of this analysis, as described previously (Section 2.2.1; Supplemental Figures S1 and S2). For each plant BNS food group, the median protein level across foods was determined. For each food within each BNS food group, RDIs were allocated based on Schedule K of Canada’s Food and Drug Regulations [39]. When an RDI was not available, the RA was used [30]. The average RDI and RA for food items within the BNS food group were determined. The protein rating was calculated by multiplying the PER by the average RDI or RA as per Canadian requirements [34].

If PER data were available, the PER method was applied to the BNS subgroup to determine eligibility for a PCC under Canadian regulations. PER values from the Canadian Food Inspection Agency were applied [30] (Supplemental Table S9). When BNS food groups contained different foods with different PERs, the mean PER was used.

When a PER was not available for a plant BNS food group, a PER was calculated based on Health Canada’s interim policy of deriving a PER from a PDCAAS. Briefly, median levels of protein and each IAA (mg/g protein) within each plant BNS group were determined. The median level of each IAA was divided by the IAA requirement (mg/g protein) for 2–5-year-old children as per the FAO’s recommendation (Supplemental Table S11) [11]. The lowest ratio was determined to be the AAS. The peer-reviewed literature was used to determine the true N digestibility coefficients of plant foods to be applied to PDCAAS assessments. When >2 true N digestibility coefficients were available for a plant BNS food group, the mean true N coefficient was used to calculate the PDCAAS for the food group (Supplemental Table S10). When ≤2 true N digestibility coefficients were available or not available for a plant BNS food group, as was the case for fruit and vegetable groups, a conservative 0.8 N digestibility coefficient was applied to the entire BNS food group based on previous analyses [40]. The PDCAAS for the BNS food group was calculated by multiplying the AAS by the N digestibility coefficient. The PER for each BNS food group was calculated by multiplying PDCAAS by 2.5 as outlined in Section 2.2.1. Once a PER was generated, a protein rating was calculated to determine the eligibility of each plant BNS food group for a PCC.

##### Eligibility of BNS Food Groups for a PCC Under US, ANZ, and EU Regulations

For the US, PDCAAS for each BNS food group was determined as previously described (Section 2.2.1) and used the same true N digestibility assumptions used in applying PDCAAS under the Canadian framework (Section Eligibility of BNS Food Groups for a PCC Under Canadian Regulations). Corrected protein was calculated by multiplying PDCAAS by the amount of protein per Canadian RA. The RDI was not used. The amount of corrected protein was divided by an assumed DV of 50 g/day as per Supplemental Figure S3.

For the ANZ PCC framework, the median total protein (g/100 g serving) was multiplied by the average RA for each plant BNS food group. Eligibility for a PCC in the EU was determined by evaluating the median protein per 100 g in the plant BNS food group. This was multiplied by the Atwater conversion factor for protein (4 kcal/g protein) and divided by the median energy per 100 g across food items in the same group.

For all calculations, identification of a BNS food group that qualified for a PCC was based on requirements for a “source of protein” claim (Table 1). However, “high source” PCC frameworks were used in a sensitivity analysis under the EU regulations.

### 2.3. Identification of Consumers and Non-Consumers of “Source of Protein” Plant Foods

Respondents from the CCHS of 2015 were characterized as “consumers” if they consumed ≥1 plant food, regardless of amount, from a plant BNS food group characterized as a “source of protein” from PCC regulations from Canada, the US, ANZ, or the EU. This study assumed that the presence of a PCC facilitated the purchase and consumption of a plant food qualifying for a PCC under a regulatory framework. Respondents who did not consume any plant food from a plant BNS food group eligible in a PCC framework were characterized as a “non-consumer”.

### 2.4. Calculating the DIAAS and the Corrected Protein Intakes in PCC Consumers and Non-Consumers

The PQ of the diets for each CCHS participant was calculated and accounted for protein complementarity from different food sources using the Digestible Indispensable Amino Acid Score (DIAAS). Consumption of protein from all BNS food groups and food items was used to determine the DIAAS of daily diets for each individual. Published in 2013 by the FAO, DIAAS utilizes updated IAA reference patterns across the lifespan (mg/g protein) and uses ileal IAA digestibility coefficients [41]. However, if an ileal digestibility coefficient is not available, true N protein digestibility is to be used [41]. This was the case for this study. A conservative true N digestibility coefficient of 0.8 was applied to all BNS food groups, based on previous analyses [40].

Briefly, for each individual, 24-h recall data were used to estimate the protein intake from each BNS food group. Protein from each BNS food group was derived from compositional data of food items from the CNF. IAA estimates for plant and animal BNS foods groups were calculated as median values across food items within the group. Daily IAA (mg/g protein) intakes from each BNS food group were summed, and each was divided by the corresponding IAA (mg/g protein) recommendation from the IAA reference scoring for older children, adolescents, and adults [41] (Supplemental Table S11). This was the appropriate reference pattern given that adults ≥ 19 years of age were used in this analysis. The IAA score was the IAA with the lowest ratio. To determine the DIAAS of diet, the IAA score was multiplied by the 0.8 true N digestibility coefficient. A DIAAS of 1.00 represents a dietary pattern meeting 100% IAA requirements. As per the standard method for calculating the DIAAS for mixed diets, DIAAS values > 1.00 were truncated to 1.00 [41].

Corrected daily protein intakes (g/day) were determined by multiplying the daily DIAAS for each individual by their total crude protein intake (g/day) based on specific foods consumed. To understand the differences in PQ and corrected protein, a DIAAS for plant and animal sources was also calculated.

### 2.5. Macronutrient and Micronutrient Intakes of Participants in Each PCC Group

Although BNS food codes were used to determine allocation to PCC groups and PQ of daily diets, all macronutrient and micronutrient intakes were based on the composition of specific foods consumed by each participant.

### 2.6. Statistical Analysis

Data cleaning and preparation were completed using R Studio version 2024.04.2 + 764, and all statistical analyses were conducted using SAS version 9.4 (SAS Institute Inc., Cary, NC, USA). To ensure national representation, estimates were weighted using the survey weights provided by Statistics Canada. Variance estimation was performed using the bootstrap balanced repeated (BRR) replication with 500 replicates. All nutrients were expressed as either a percentage of energy (%E) or per 1000 kcal to adjust for energy. To satisfy the normality requirement of statistical tests, the values of the nutrients were transformed to approximate a normal distribution using the Box–Cox method.

Participant characteristics are described using mean and standard error (SE) for continuous normally distributed variables and number and frequency for categorical variables.

Three separate analyses were used to compare PQ, protein quantity, and nutrient intakes (macronutrient and micronutrients) between consumers and non-consumers of plant protein foods meeting a PCC in Canada compared to either the US, ANZ, or the EU. Participants in each of the three analyses were classified as meeting the PCC in one of the comparison frameworks, both comparison frameworks, or neither of the comparison frameworks (non-consumers). Comparisons were assessed using ANCOVA with post-hoc Bonferroni adjustment for multiple comparisons (PROC SURVEYREG). All models were adjusted for the following variables: misreporting status (EI/TEE), age, sex, smoking, self-perceived health, blood pressure, diabetes, heart disease, cancer, osteoporosis, education, physical activity, income, BMI, immigrant status, and weekend reference day, as previously described [24]. Two-tailed *p*-values at <0.05 were considered statistically significant.

Given that the EU’s PCC framework is based on energy, substantially more plant BNS food groups qualified for a “source of” PCC compared to Canada, ANZ, and the US. A sensitivity analysis was completed to compare the PQ, crude and corrected protein quantity, and nutrient intakes (macro and micronutrients) between consumers of plant foods eligible for a “high source” PCC according to the EU, “source of protein” PCC in Canada, and non-consumers. The sensitivity analysis was limited to the EU framework because the higher threshold for “high source of protein” claims in the US and ANZ limited the number of plant BNS food groups that met the PCC requirements.

## 3. Results

### 3.1. Sample Characteristics

Table 2 summarizes the sample-weighted participant demographics across the adults included in this study (n = 11,817). Females (51%) and males (49%) were evenly represented among participants with a mean age of 49 (±0.26) years old, BMI of 27.7 (0.1) kg/m^2^, and with over half reporting post-secondary education (57%). Most participants reported being non-smokers (81%) and had not been diagnosed with high blood pressure (75%), heart disease (93%), diabetes (91%), cancer (97%), or osteoporosis (50%).

### 3.2. Plant Food Groups Eligible for a PCC by Regulatory Framework

**Figure 3:**
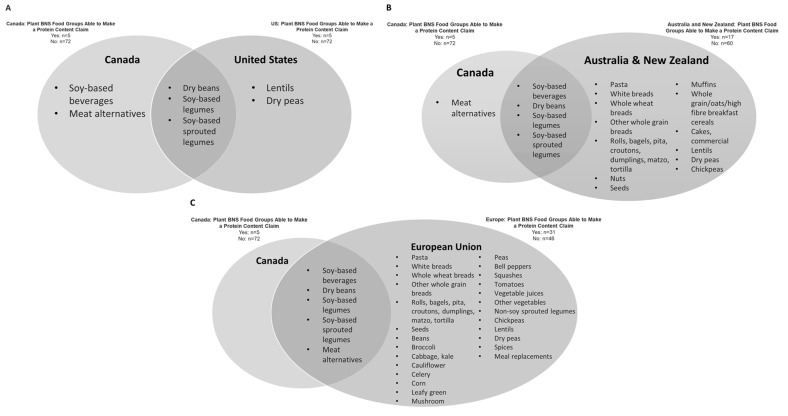
Comparison of plant BNS food groups that qualified for a “source” of protein content claim across regions. (**A**) Canada vs. the US; (**B**) Canada vs. ANZ; (**C**) Canada vs. the EU. Areas of overlap represent plant BNS food groups that qualified for a PCC in both regions. ANZ, Australia and New Zealand; EU, European Union; PCC protein content claim.

Of the 163 BNS food groups, 27 were negligible protein sources or did not provide information on the protein and IAA of foods, 59 were animal-based protein sources, and 77 were characterized as containing plant protein or a mixture of plant and animal protein foods (Supplemental Table S12). Figure 3 shows that under Canadian regulations and the interim policy for a PCC at the time of the analysis, five plant BNS food groups met the minimum requirements for a PCC: plant-based beverage (soy), dry beans, soy-based legume, soy-based sprouted legume, and meat alternatives. Similarly, five plant BNS food groups qualified for a PCC under the US framework. Nuts and seeds did not qualify for a PCC in either Canada or the US. Dry beans were the only pulse that was eligible for a PCC in Canada, while in the US, lentils and dry peas qualified for a PCC.

Using the ANZ and EU PCC frameworks, 17 and 31 plant BNS food groups met the criteria for a “source of protein” PCC, respectively. For both regions, soy and all categories of pulses (dry beans, chickpeas, dry peas, and lentils) were eligible for a PCC. ANZ was the only framework where nut and seed BNS food groups met a “source of protein” PCC. More grain-based food groups were eligible for a PCC in the EU and ANZ compared to both Canada and the US. In the EU, given that the PCCs are based on energy from protein content per 100 g, a substantial number of vegetable BNS food groups also qualified for a PCC compared to other regions.

When the eligibility for a PCC for the EU was re-evaluated using the cutoff for a “high source of protein” at 20% energy from protein per serving, the number of plant BNS food groups that qualified for the claim decreased from 31 to 18 (Supplemental Figure S4).

### 3.3. Comparison of Protein Quality and Quantity of Diets Corresponding to Consumption of Plant Foods That Qualified for a PCC According to Different Regulatory Frameworks

#### 3.3.1. Protein Quantity and Quality of Diets: Canada vs. The US PCC Frameworks

Protein and PQ comparisons between Canada and the US PCC regulatory frameworks are summarized in Table 3. In total, 10,405 respondents were categorized as non-consumers and did not consume any plant protein foods from a plant BNS food group that met a PCC in either Canada or the US. Conversely, 227 and 164 participants consumed ≥1 plant protein food that qualified for a Canadian or US PCC, respectively. A total of 1021 participants consumed ≥1 plant protein food that qualified for a PCC under both Canadian and US regulations.

There was no difference in total or percent energy from protein across groups. Animal and plant protein intakes differed across groups (*p* = 0.001 and *p* < 0.00, respectively). Plant protein intake was higher in the US (51.6 g/d) and the Canada + US PCC (35.5 g/d) groups compared to non-consumers (26.1 g/d). Animal protein intake was similar between non-consumers (52.1 g/d) and the Canada PCC group (58.3 g/d) and higher (*p* < 0.05) compared to the other two PCC groups.

The DIAAS was >0.94 and similar across all groups. DIAAS for plant protein was significant across groups (*p* < 0.001) and was higher for PCC consumers (0.649–0.766) compared to non-consumers (0.591). Plant protein DIAAS for the US and Canada + US PCC groups were both higher (*p* < 0.05) than Canada’s PCC and non-consumer groups. Corrected total protein intake was similar across all groups, while corrected animal protein and plant protein were significant across groups (*p* = 0.001 and *p* < 0.001, respectively). Corrected plant protein intake was higher in the Canada PCC (21.5 g/d), US PCC (38.8 g/d), and Canada + US PCC (25.1 g/d) groups compared to non-consumers (15.3 g/d).

#### 3.3.2. Protein Quantity and Quantity of Diets: Canada vs. The ANZ PCC Frameworks

Protein and PQ comparisons between Canada and ANZ PCC regulatory frameworks are summarized in Table 4. Non-consumers of plant BNS food groups that qualified for a PCC from Canada or ANZ accounted for 991 respondents. Fewer individuals (n = 1245) consumed plant foods that qualified for a PCC in Canada and ANZ. Three individuals consumed a plant protein food that qualified for a PCC in Canada only, which corresponded to consumption of the Meat Alternatives BNS food group. The ANZ PCC group accounted for 9578 individuals.

There was a difference in total protein intake across groups (*p* = 0.046). However, there were no pairwise differences between any groups, and there was no difference in percent energy from protein across all groups. Animal and plant protein intakes differed across groups (*p* = 0.024 and *p* < 0.001, respectively). Non-consumers had higher (*p* < 0.05) intakes of animal protein (53.3 g/d) compared to the Canada + ANZ PCC group (49.0 g/d). Conversely, plant protein intake was significantly higher in all PCC groups compared to non-consumers.

All DIAAS for total protein were >0.95 across all groups, and there was a significant difference across groups (*p* = 0.005). However, there were no significant pairwise differences between any groups. There was a significant difference across groups for plant foods DIAAS (*p* < 0.001), where the ANZ PCC group had the lowest plant protein DIAAS (0.589) compared to non-consumers (0.649), Canada (0.685), and Canada + ANZ (0.694) groups. There was no difference in total corrected protein across all groups. The corrected plant protein intake was significantly different across groups (*p* < 0.001), where the ANZ (16.3 g/d) and Canada + ANZ (24.5 g/d) PCC groups had higher corrected plant protein intakes compared to the non-consumers (10.4 g/d) groups.

#### 3.3.3. Protein Quantity and Quality of Diets: Canada vs. The EU PCC Frameworks

Protein and PQ comparisons between Canada and the EU PCC regulatory frameworks are summarized in Table 5. Non-consumers accounted for 308 respondents. None of the respondents consumed a plant BNS food group that qualified for a PCC in Canada only, while 1248 individuals consumed “source of protein” foods from an eligible plant BNS food group in Canada and the EU frameworks. The EU PCC group had the most allocated individuals (n = 10,261).

There was a difference in total protein intake across groups (*p* = 0.021); however, there were no pairwise differences and no difference in percent energy from protein across groups. Animal and plant protein intakes differed across groups (*p* = 0.028 and *p* < 0.001, respectively). Plant protein was significantly higher in the EU PCC group (26.9 g/d) and Canada + EU PCC group (35 g/d) compared to non-consumers (14.7 g/d).

All DIAAS were >0.95 with no significant difference across groups. There was a significant difference across groups for DIAAS from plant foods (*p* < 0.001), where the DIAAS was significantly higher for the Canada + EU PCC (0.694) group compared to the EU PCC group (0.594) and non-consumers (0.592). The corrected protein intakes were significantly different across groups (*p* = 0.015); however, there were no significant differences in pairwise comparisons or in corrected protein intakes expressed as percent energy (*p* = 0.998). The corrected animal and plant protein intakes were significantly different across groups (*p* = 0.033 and *p* < 0.001, respectively). Corrected plant protein intakes in the EU (15.9 g/d) and Canada + EU PCC groups (24.4 g/d) were significantly higher compared to non-consumers (9.0 g/d).

The sensitivity analysis that compared the EU’s “high source of protein” PCC threshold (≥20% energy from protein per serving) and Canada’s base PCC criteria resulted in more non-consumers (n = 1885) and fewer EU consumers (n = 8684) but demonstrated similar results (Supplemental Table S13).

### 3.4. Energy and Non-Protein Macronutrients and Micronutrient Intakes of Diets Corresponding to Consumption of Plant Foods That Were Eligible for a PCC Across Different Regulatory Frameworks

#### 3.4.1. Energy and Nutrient Intakes of Diets: Canada vs. The US PCC Frameworks

Energy and nutrient intake comparisons of consumers of plant foods according to the Canada and US PCC regulatory frameworks are summarized in Table 6. There was no significant difference in total energy intake across groups. The US PCC group had higher intakes of carbohydrates, total fibre, and folate and lower intakes of saturated fat and alcohol compared to the other PCC groups and non-consumers (*p* < 0.05). The US and Canada + US groups had higher folate, iron, and magnesium, while the Canada + US group had higher linolenic acid, cholesterol, potassium, and zinc. The Canada and Canada + US groups had higher calcium, and the Canada group had higher PUFA, vitamin D, and phosphorus compared to non-consumers. Non-consumers had higher niacin compared to the US and Canada + US groups. No differences were observed in other nutrients in pairwise comparisons.

#### 3.4.2. Comparison Between Canada and ANZ PCC Regulations

Energy and nutrient intake comparisons between Canada and ANZ PCC regulatory frameworks are summarized in Table 7. Energy intake differed across groups (*p* < 0.001), where non-consumers had lower total energy intake compared to the PCC groups.

Consumers in the ANZ and Canada + ANZ PCC groups had higher intakes of total fibre, thiamin, and iron, with lower vitamin B6, compared to non-consumers (*p* < 0.05). Consumers in the Canada + ANZ PCC group have lower cholesterol, higher folate, magnesium, and calcium, but higher sodium compared to non-consumers, and higher zinc compared to other PCC groups (*p* < 0.05). All PCC groups had higher PUFA, linoleic, and linolenic acid compared to non-consumers (*p* < 0.05).

The ANZ group had higher niacin and lower vitamin C and potassium compared to the Canada + ANZ groups. The Canada group (n = 3) had higher total fat and saturated fat and lower vitamin A compared to all groups, higher sugar compared to the ANZ group, lower riboflavin compared to other PCC groups, and lower alcohol compared to non-consumers and the ANZ group.

#### 3.4.3. Comparison Between Canada and EU PCC Regulations

Energy and nutrient intake comparisons between Canada and the EU PCC regulatory frameworks are summarized in Table 8. Energy intake differed across groups (*p* < 0.001), where non-consumers had lower total energy intake compared to the EU and Canada + EU PCC groups.

The Canada + EU and EU groups demonstrated higher intakes of total fibre, fat, linolenic acid, thiamin, folate, vitamin C, and iron, as well as lower (*p* < 0.05) sodium compared to the non-consumer group (*p* < 0.05).

The Canada + EU group consumed more PUFA, linoleic acid, calcium, magnesium, phosphorus, and potassium, and lower cholesterol and niacin compared to non-consumers (*p* < 0.05). The EU group had lower intakes of calcium, magnesium, and zinc compared to the Canada + EU group (*p* < 0.05).

The sensitivity analysis that compared the EU’s “high source of protein” PCC threshold (≥20% energy from protein per serving) and Canada’s base PCC criteria demonstrated similar results with the following exceptions: intakes of total fibre and fat were no longer different between Canada + EU and EU compared to non-consumers and intakes of PUFA, linoleic acid and niacin were no longer different between Canada + EU and non-consumers, and intakes of calcium and magnesium were significantly greater in the EU group compared to non-consumers (Supplemental Table S13).

## 4. Discussion

The present study compared the protein quantity, PQ, corrected protein quantity, and other macronutrient and micronutrient intakes of Canadians, as the consumption of plant foods was modelled to be based on the utilization of regional PCC regulatory frameworks. Results showed that total protein intake and percent energy from protein were relatively consistent across non-consumers and plant PCC groups. Although the application of DIAAS to diets revealed some differences in PQ across groups, there were no pairwise differences, and the PQ of all diets was >0.94 despite varying inclusiveness of plant protein foods between regional PCC criteria. Corrected protein intake was also consistent across PCC groups at 74–89 g/d or 16%E, similar to non-consumers at 68–78 g/d or 17% E. PCCs for plant foods facilitated higher intakes of corrected plant protein compared to non-consumers without resulting in important changes in total protein intakes or PQ. These changes were seen while generally increasing positive nutrients (dietary fibre, iron, magnesium, calcium, folate, potassium) and decreasing negative nutrients (saturated fat and cholesterol). In some instances, there were decreases in niacin and increases in energy and sodium.

### 4.1. Findings of Protein Quantity and PQ in the Diet in Relation to Existing Literature

Our findings show some differences with existing data from Canada and the US. Previous PQ assessments of the CCHS and NHANES datasets have shown that when >50% of protein was from plant foods, the PQ of diets significantly decreased [40,42]. However, this was due to a lack of variety for plant protein and protein complementarity in diets, where most plant protein was from cereal grains. Cereals represent the primary source of plant protein in diets globally, including Canada and the US, due to high rates of production and abundance in food systems [43]. Intakes of plant protein foods, such as legumes, nuts, and seeds, are comparatively low [44,45]. Replacing 50% of IAA from cereals with pulses has been shown to increase the PDCAAS of diets by 10% [42]. The present study assessed diets using a food-based approach, identifying those foods meeting a PCC in contrast to using plant protein intake alone, which may be driven by high consumption of one type of plant protein.

One reason a food-based approach for assessing PQ may better capture overall PQ is that it allows for contributions from other food sources of protein. Complementarity has been shown to be a key attribute to meeting IAA as plant proteins become the dominant source of protein in dietary patterns, but it is less important when plant protein represents a modest contribution of protein to the diet [46]. The observed high DIAAS (>0.94) and corrected protein intakes (>70 g/d) across all PCC groups demonstrate protein complementarity.

### 4.2. Findings of Energy and Nutrient Intakes in the Diet in Relation to Existing Literature

Our findings regarding nutrient intakes show similarities with existing data from Canada. Other analyses of CCHS data has shown that, while protein content decreased, a diet characterized by 50–75% plant protein foods led to higher intakes of carbohydrate, dietary fibre, folate, magnesium, iron, PUFA and linoleic acid and lower intakes of saturated fat and cholesterol compared to diets with average plant protein intakes (25–50% plant protein) [47]. Again, while these data are largely driven by cereals, they may be important contributors to increasing positive nutrients and reducing negative nutrients. While legume, nut, and seed intakes are low [48,49], they may also be contributing to these nutrient differences.

Total energy and sodium intakes were higher in consumers across some PCC groups compared to non-consumers. However, all analyses were adjusted for BMI and energy (expressed as g/1000 kcal or %E), thus the observed nutrient differences were independent of energy.

### 4.3. Implications

Most Canadians consume diets that contain between 25 and 49% plant protein [47], with mean intakes of 30–36% [40,44]. Designated plant protein foods that derive protein from legumes, nuts, and seeds contribute only 5% of protein to Canadian diets [44]. PCCs are a critical tool that consumers use for identifying protein foods in the retail environment [50]. A systematic review and meta-analysis demonstrated that the odds of selecting a product with a health claim were 75% higher than identical food items without these claims [51]. The effects were larger for foods with claims on products categorized as ‘Beans, Pulses, Fish, Eggs, Meat and other Proteins’, where there was a 2.4-fold increase in the odds of purchasing these foods with claims compared to without claims [51]. Compared to other labelling claims, “source of protein” claims have also been shown to be the most understood and have the most value for consumers when deciding to try a food product [52]. Thus, PCCs can present an opportunity to support greater adherence to CFG.

The use of PQ as part of PCC regulations in Canada and the US is based on ensuring that all IAAs required for growth and/or maintenance are attained when digestibility is considered. With some exceptions, such as soy protein, plant proteins generally have lower levels of IAA compared to animal foods. The lower PQ of plant foods does not negate their contribution to protein and IAA intakes and provides the opportunity for protein complementarity [53]. This reflects how proteins are consumed as part of a healthy dietary pattern.

Although PQ in the context of PCC regulations for Canada and the US is a discrete assessment of individual foods, there is some acknowledgement or semblance of protein complementarity within the Canadian PCC framework, with special consideration given to milk and breakfast cereal. Canadian regulations stipulate that PQ calculations for breakfast cereals can include 125 mL milk [29]. On their own, this volume of milk and most breakfast cereals would not qualify for a PCC in Canada, where regulations acknowledge that these foods are consumed together. Embedding PQ in PCC regulations in Canada limits the utility of PCC for plant foods.

Another disconnect between the promotion of plant protein in dietary guidance [54] and the ability to communicate the protein attributes of foods to consumers in Canada is the limitation in food sources of plant proteins meeting a PCC in Canada. While in ANZ and EU, soy-based foods, beans, lentils, chickpeas, dry peas, nuts, and seeds met the criteria for a PCC, in Canada, only some soy-based foods qualified for PCC. The inability of beans and other pulses to qualify for a PCC under the Canadian regulations is due to several methodological considerations embedded in Canada’s PCC regulations. First, the use of PER inflates sulfur amino acid requirements because of the heightened needs of rodents. Pulses are known to have relatively lower levels of sulfur amino acids than the requirements. Second, Canada’s regulatory requirement to apply RDI for PCC assessments. In this study, the RDI for baked beans (250 g) was applied to the BNS “dry bean” subgroup. For other pulses, an RDI does not exist. Thus, as per Canada’s policy, the RA of 125 mL was used for the PQ calculation. Thus, rather than substantial differences in IAA composition, the qualification of dry beans for a PCC within the Canadian PCC framework was due to the higher amount of beans that is assumed to be consumed over the course of the day, compared to a “serving” for other pulses. Protein analyses of samples of cooked pulses have also highlighted challenges with meeting PQ thresholds to carry a PCC in Canada and the US [55]. It is valid to question the utility of the RDI for PCC eligibility, as it may not reflect the amount of food that is consumed over the course of a day by a consumer. PCC assessments are based on the regulated serving sizes (RACC) in the US and serving sizes in ANZ and the EU.

Both traditional and innovative plant protein foods can contribute to protein and IAA intakes [46,56,57,58]. Most Canadians will remain omnivores and attain protein from both animal and plant sources [59]. Enhancing Canadians’ ability to identify more plant protein foods at the point-of-purchase may contribute to intakes of IAA in combination with plant and animal foods consumed throughout the day. Previous analyses have shown that when measured across the entire day or as the sum of PQ assessments for eating occasions, the daily PQ of diets does not change [40]. To date, there is no evidence to suggest that PCC frameworks from ANZ or the EU are facilitating insufficient protein consumption. Canada’s latest food guide encourages the selection of protein from plant foods more often. However, with only 5% of total protein coming from legumes, nuts, and seeds [44], Canadian diets are not aligned with the 2019 CFG [1]. Therefore, there is an opportunity for a broadening of PCCs to support the identification and purchasing of good sources of plant proteins by Canadians to better align with recommendations in CFG.

Protein quality is an important metric of protein intake and adequacy in diets. However, examination of the PQ of foods in isolation when individuals are consuming mixed diets does not provide a wholesome picture of protein adequacy and how protein and IAA are consumed. The application of DIAAS to dietary patterns across PCC groups in this study supports the paradigm of protein complementarity within a dietary pattern. DIAAS across PCC groups and non-consumers was high at >94%. Given that the majority of Canadians (84–95%) are omnivores [59,60] and the allocation to a PCC group was based on consuming at least one plant food that aligned with PCC criteria for a region, individuals in PCC groups also consumed animal foods (meat, eggs, dairy, etc.) as part of their dietary pattern. Thus, it is unlikely that the removal of PQ would affect protein intake and adequacy in Canadian diets. An analysis of Dutch diets demonstrated that protein adequacy remained sufficient for most individuals when the replacement of meat with meat analogues was modelled [61]. Furthermore, rather than PQ, lysine intake and digestibility had greater effects on protein adequacy. A modelling analysis of French diets demonstrated that, when <50% of the protein in diets is plant protein, protein intake rather than PQ was a greater predictor of protein adequacy [46]. At >70% plant protein, the same study showed that protein variety from legumes, nuts, and seeds was most important to meet protein and lysine requirements through complementarity [46]. This case highlights how the ability to label such foods as sources of protein would support meeting protein needs. It is important to note that this study used the minimum criteria for identifying plant BNS food groups that qualify for a PCC. In the application of PCC in the marketplace, animal-sourced proteins would qualify for higher-tier PCCs using “high-” or “excellent source of protein” to differentiate from most plant protein foods (Table 1).

Although DIAAS was applied to assess the PQ of diets in this study, DIAAS has not been applied by any country or region as a framework to support PCC. Developed and endorsed by an FAO consultation, DIAAS addresses challenges inherent to the PDCAAS methodology [41]. Although a more accurate measure of PQ than PDCAAs, one identified challenge with the use of DIAAS as a proposed regulatory framework for PCC is the minimum DIAAS threshold of 0.75 (75%) to qualify for a protein claim. An analysis by Sa et al. [62] demonstrated that across lentil samples, none would qualify for a PCC using DIAAS because of the 75% cutoff. Similar to the current application of PQ in Canada and the US, this would significantly disadvantage plant protein foods from making a PCC and would not consider the utility of plant protein in the context of a dietary pattern. New methods of PQ have been developed that borrow elements of PDCAAS or DIAAS methods, but also simultaneously incorporate other elements of PQ such as total protein (nitrogen) and dispensable amino acid requirements [63,64].

Given the breadth of protein foods in diets, it has been speculated that removal of PQ from PCC assessments could cause an influx of food innovation that uses high protein ingredients (i.e., collagen) devoid of IAA to falsely inflate the protein credentials of foods [16]. While possible, the concern is speculative and is not a known practice that food manufacturers engage in within the regions examined in this study. Moreover, the use of these ingredients, such as collagen, in food formulations at a level that would enable a PCC would have hedonic consequences for food innovation. It could be reasonable to regulate against the use of a PCC on a food when a single ingredient devoid of IAA is used to inflate the protein content of the food in question. Given the amount of protein consumed in developed countries, including Canada, it is unlikely that consumers would not meet protein requirements, even when proteins of lower digestibility were consumed [16].

In this study, more inclusive PCC frameworks from ANZ and the EU also identified many cereal-based BNS food groups as “sources of protein”, while these did not meet PCC frameworks in Canada and the US. Given that the EU’s PCC framework is based on energy from protein, vegetables and spices were also identified as plant food groups that could carry a PCC. Cereals are not considered to be a protein food in dietary guidelines, and it could be suggested that the use of a PCC on some cereal products could further enhance their intake rather than the intakes of foods characterized as plant-based proteins, such as legumes, nuts, seeds, or their derivatives. This is a reasonable concern. However, at a 5 g protein per serving as the minimum level of protein for a PCC in ANZ, these types of foods could be contributing significant protein and IAA in the context of a dietary pattern. For vegans, cereals would be a valuable source of protein in diets, in combination with designated plant proteins such as legumes, nuts, seeds, and protein analogues to support complementarity. Furthermore, wheat flour is subject to mandatory fortification for iron and folate in Canada, which could also be important for vegan and some vegetarian diets [65]. Any major changes to a diet, such as transitioning to a vegan or vegetarian diet, should be conducted in consultation with a healthcare provider. Any revision to a PCC framework may consider reflecting on protein content per serving.

The broader impact of PQ on national food policies also requires consideration. CFG and other guidelines increasingly promote plant protein from the perspective of nutritional adequacy and reducing the environmental impact of diets [66,67]. Katz et al. [68] previously explored nutrition, health, and climate change as a conceptual framework of PQ. When PDCAAS was combined with scores corresponding to health and the environmental impact to ascertain a measure of PQ, plant protein had a higher PQ than animal protein-based foods. This type of analysis is only relevant if PQ would be applied to the broader implications of protein choices. In the US, environmental sustainability is not considered a pillar of dietary guidance. Conversely, CFG recommends plant-based protein “more often” for dietary, health, and environmental attributes. It is not suggested herein that the scope of current PQ measures be expanded at this time, but we acknowledge that there are broader implications for labelling and effects on the food system. While it is reasonable that low- and medium-income countries may benefit from increasing consumption of animal proteins, developed countries, such as Canada, may benefit from a reduction on a population level, with exceptions for specific vulnerable groups (e.g., infants, elderly).

In addition to sustainability, social equity and rising food prices remain a concern for Canadians. Food insecurity is also a significant challenge in Canada, where consuming less meat is a strategy to reduce household food costs [69,70]. It is well known that traditional plant protein foods cost less than animal-based products [71]. This is caveated with animal protein analogues demonstrating higher prices compared to animal-based products in Canada [72]. These factors are relevant when considering the criteria for making PCCs in Canada and facilitating sufficient intakes of protein by Canadians.

Prior to 2024, the Canadian PCC regulatory framework had not been revised since the early 1980s; Health Canada recently released a call for evidence to support a modernization and re-evaluation of Canada’s PCC regulatory framework [73]. In demonstrating no clinically relevant differences in PQ or protein quantity in consumers of foods meeting PCCs across frameworks in this study, there is support for broadening the current PCC framework in Canada to support the nutrient intake of non-consumers of plant protein foods. While PQ assesses the individual food by its amino acid and digestibility, it can disqualify protein foods that facilitate the inclusion of plant foods linked to reduced risk of cardiometabolic disease and prevent protein complementarity in the context of the dietary pattern. These data suggest the following: 1. Canada’s approach to PCC is detracting from the overarching purpose of nutrient content claims, given the variety of food available in developed food environments. 2. A PCC framework in Canada that aligns with the EU and ANZ could assist with the adoption of a more inclusive dietary pattern for a substantially greater proportion of individuals that is more nutrient-dense while meeting protein and IAA intakes. A re-evaluation of Canada’s PCC regulatory framework may play a key role in encouraging individuals to increase the array of plant protein foods in their diets, as well as leverage new plant-based food innovation that can assist with dietary behaviour change.

Concerns that increasing plant protein in the diet may impact protein quantity and PQ are not reflected in the present data. Furthermore, recent analyses show that protein inadequacy is not a major health concern in Canada, with only 3% of Canadians consuming protein below the estimated average requirement [44]. The prevalence of protein inadequacy in females > 71 years old was 9.8%. This data is mirrored by analyses of national survey data (NHANES) in the US [74,75]. The protein data in the present study demonstrate how supporting the selection of plant protein foods may play a role in a varied diet, which can support the intake of other positive nutrients.

Lastly, our findings demonstrated that consumers of plant protein foods that met the criteria for inclusive PCC frameworks had higher intakes of several positive nutrients commonly under-consumed by >20% of Canadian adults in specific age segments [76], such as dietary fibre, calcium, and iron, as well as folate and magnesium compared to non-consumers. Therefore, increasing the promotion of plant protein foods can support Canadians in meeting the requirements of key nutrients of concern. Furthermore, increasing consumption of plant protein foods as a substitution for animal protein foods can play an important role in the cardiovascular health of Canadians by improving blood lipids [77] and glycemic control [78]. More broadly, diets rich in plant proteins are associated with improved cardiometabolic health, reduced all-cause mortality, and a positive impact on the global climate crisis [8,79,80].

For some comparisons, there were increases in sodium intakes for PCC groups compared to non-consumers. Although not significant, substantially more sodium was consumed by the Canada PCC group (2035.8 mg/d) versus non-consumers (1495.5 mg) in the Canada and ANZ comparison. This could have been due to meat alternatives being the only plant BNS food group that qualified for PCC for the Canada PCC group, which are known to have high levels of sodium as a flavor enhancer. The EU PCC and Canada + EU PCC groups also had significantly higher sodium intakes compared to non-consumers. Higher sodium in the EU PCC group could have been from the inclusion of bread-type BNS food groups, spices, and vegetables as PCC foods. Breads can contain significant levels of sodium for flavor and dough formation, while spices and canned vegetables may contain added salt. Health Canada has identified various categories of bread as strategic targets for reducing sodium intake from processed food in Canada [81]. However, mean sodium intakes were below the chronic disease risk reduction threshold of 2300 mg/day [82] across all PCC and non-consumer groups.

It is worthwhile to note that in December 2024, Canada’s Food and Drug Regulations were changed, where Health Canada officially adopted PDCAAS as an additional method to support eligibility for a PCC [29]. The PER method can still be used. Canada’s application of PDCAAS differs from that of the US and the interim policy used in this analysis. Rather than using PDCAAS to calculate a PER (PDCAAS × 2.5 = PER) [30], Canada’s new regulation allows PDCAAS to calculate a protein rating by multiplying PDCAAS by the amount of protein per the RDI of a food [83]. PCC protein rating cut-offs for the new PDCAAS method are 8 and 16 for “source” and “high source” claims, respectively [29]. While these protein rating cut-offs differ from the original PER cutoffs (20 and 40, respectively), the new PDCAAS framework for Canada was designed to yield the same result as the PER PCC method [38]. For this study, it is possible that the new application of PDCAAS may have yielded different results when a PER value was not available. However, because the new regulatory framework was developed to give similar results for a “source of protein” claim regardless of whether the PER or PDCAAS method is used to derive the protein rating, it is unlikely that the results of this study would demonstrate more plant BNS food groups qualifying or PCC under the new Canadian framework.

### 4.4. Strengths and Limitations

This research study has several strengths. These analyses used Canada’s most recent large, nationally representative CCHS dataset. Key information, such as diet, BMI, physical activity, health status, and socioeconomic factors, was captured using validated tools, which helps minimize the potential for information bias. Statistical analyses were adjusted for misreporting status, reducing the possible influence of recall bias. Our adjustments also included several covariates, which improved the precision of the research findings.

This study has some limitations that should be considered in the interpretation of the research findings. First, the study’s sampling restrictions limit the generalizability of results as some demographic groups were excluded from the analysis. Second, dietary data were collected through a single-day 24-h food recall, which may not accurately represent the usual intake of episodically consumed foods. This could potentially lead to underestimation or overestimation of food consumption, although all nutrients were energy-adjusted. Furthermore, while nutrient intakes demonstrated significant differences between groups, some of these differences may not be clinically significant in the context of nutritional adequacy.

Third, PDCAAS calculations for designating plant BNS Food groups as “sources of protein” for Canada (when a published PER was not available) and the US used 0.8 as the true N digestibility coefficient when insufficient digestibility data were available. Similarly, a 0.8 digestibility coefficient was applied to all foods when determining the total daily DIAAS. The application of an estimated digestibility coefficient could have introduced bias into the analysis. However, the estimate used in this study was considered to be conservative given that most protein foods have digestibility coefficients that exceed 0.8. For assessment of PDCAAS under the US or interim PCC framework for Canada, the same conservative coefficient was used for plant foods when a mean estimate could not be derived from the literature. Although proteins from different foods differ in digestibility, most animal proteins have digestibility values > 95%. While more variable, protein digestibility coefficients for plant foods can also be quite high [84]. In vivo digestibility coefficients for most pulses, nuts, cereals, and almonds have been shown to be >0.85 [55,85,86]. Isolated protein from pulses can have digestibility coefficients > 0.90% [87,88]. The use of true N digestibility coefficients has been criticized because the effects of the microbial environment in the large intestine are not accounted for and can inflate digestibility values. This has led to a newer consensus that the ileal digestibility coefficient for PQ should be determined as described by the DIAAS method [41]. However, while ileal digestibility coefficients for each IAA for each protein are not available, true N ileal coefficients for plant protein, for the most part, also remain rather high at >85% [84]. Thus, it is reasonable that the PQ and corrected protein results of this study would not substantially change if more specific ileal and IAA digestibility coefficients for each food, particularly plant protein foods, were available. Rather, given the conservative digestibility estimate used for total dietary analysis of PQ, it is also plausible that PQ would increase if actual digestible coefficients were applied to the calculations. Thus, PQ assessments were likely underestimated. Nevertheless, a sensitivity analysis applying alternative digestibility coefficients to test effects on the protein intake, PQ, and nutrient intake would be beneficial.

Fourth, the application of Health Canada’s interim policy for determining the protein rating of plant BNS food groups could have introduced potential bias and errors in PQ assessments. These same challenges would also manifest in the application of the interim PCC framework within Canada’s food system. As previously discussed, Health Canada has since changed the way in which PDCAAS is used to validate PCC in Canada, where challenges persist. The application of the US PCC framework in this analysis also supports the identification of fundamental challenges with how PQ is applied to PCC frameworks. Few plant BNS food groups qualify for PCC claim, and a relatively small sample of individuals consumed plant foods eligible for a PCC in Canada and the US relative to ANZ and EU PCC groups.

The low consumption of several plant sources of proteins, such as lentils, chickpeas, beans, soy, and meat alternatives, limited the ability to observe the impact of higher intakes of these foods as protein sources on PQ, protein quantity, and nutrient intakes.

Over the last decade, there has been a substantial increase in investment in the development of innovative plant protein foods across various food platforms. The use of the 2015 CCHS from nearly a decade ago does not capture the full impact of innovative plant foods and ingredients. This challenge was also recently highlighted by the 2025 Dietary Guidelines Advisory Committee to inform the 2025–2030 Dietary Guidelines for Americans [89]. However, historical data from other datasets, such as NHANES, demonstrate that protein intake is fairly stable over time [90]. The CNF also had some limitations in the number of foods with data on IAA, where some foods were removed from the dataset prior to determining which BNS food groups qualified for a PCC for Canada, the US, ANZ, and the EU. This also limited the foods included in some categories (e.g., meat alternatives) where data was only available for plant-based bacon and sausage substitutes.

This study evaluated the PQ and nutrient intakes of adults ≥ 19 years and older. Similar analysis in children, adolescents, and perhaps older adults could be useful.

Lastly, given the use of sole sources of nutrition, the IAA requirement, with consideration given to digestibility for infant formula and, perhaps, other infant foods, remains a reasonable and regulated approach to ensuring foods meet their protein needs.

## 5. Conclusions

The present study demonstrates that the use of less restrictive regulatory frameworks for PCC used in ANZ and the EU did not substantially affect protein intake or the PQ of Canadian diets in adults. The use of PQ to underpin PCC assessments limited the number of foods that could be identified as a “source of protein” in Canada. Generally, PCC groups had increased intakes of positive nutrients and reduced negative nutrients compared to non-consumers. These results suggest that more inclusive regulatory frameworks that only use absolute levels of protein to support PCCs could support increased intake of food sources of plant proteins in alignment with Canada’s Food Guide. They may also support choosing a greater variety of foods, facilitating IAA complementarity, as well as supporting Canadians to meet their nutrient intakes and better overall health. Future research could employ a more specific approach to assessing the effects of PCC on PQ and nutrient intake by assessing the eligibility of specific foods for a PCC, rather than using plant protein-based food categories. The application of newer measures of PQ that consider IAA complementarity and protein requirements would also be useful in addition to assessments across population subgroups, such as by age and sex.

## Figures and Tables

**Table 1:** Summary of protein content claim criteria for Canada, the United States, ANZ, and the EU.

Protein Content Claim Criteria	Canada [29,30]	United States [13,31]	Australia and New Zealand [14]	European Union [15]
Method of Determination	Protein Quantity and Quality (PER and PR)	Protein Quantity and Quality (PDCAAS)	Protein Quantity	Protein Quantity
“Source” of Protein Content Claim Threshold ^1^	Protein rating ≥20 per RDI or RA ^3^	Corrected protein ≥10% DV ^4^ per RACC	≥5 g of protein per serving	≥12% of energy per serving
“High Source” of Protein Content Claim Threshold ^2^	Protein rating ≥40 per RDI or RA ^3^	Corrected protein ≥20% DV ^4^ per RACC	≥10 g of protein per serving	≥20% of energy per serving

Abbreviations: DV, daily value; PDCAAS, Protein Digestibility Corrected Amino Acid Score; PER, Protein Efficiency Ratio; PR, protein rating; RACC, reference amount customarily consumed; RA, reference amount; RDI, reasonable daily intake. ^1^ Canada: “Source “or “Good” or “High Source” of protein; United States: “Good Source of protein”; ANZ: “Contains protein”; EU: “High source of protein”. ^2^ Canada: “Very high” or “Excellent source” of protein; United States: “High” or “Excellent source” of protein; ANZ: “Good Source;” EU: “High Source of protein”. ^3^ Canada: When an RDI is not available, the RA is used. ^4^ US: The DV for protein is 50 g/day.

**Table 2:** Demographic information of the adults (≥19 years) included in this study (n = 11,817).

	n ^1^	Percent
**Age, years**	49 ± 0.26 **^1^**	
**Sex**		
Male	5670	51%
Female	6147	49%
**Smoking behavior**		
Daily	1749	15%
Occasionally	540	5%
Not at all	9519	81%
Do not know	1	0%
Not stated	8	0%
**Self-perceived health**		
Excellent	2209	19%
Very good	4391	37%
Good	3705	31%
Fair	1178	10%
Poor	321	3%
Do not know	12	0%
Refusal	1	0%
**Has high blood pressure?**		
Yes	2888	24%
No	8899	75%
Do not know	21	0%
Refusal	8	0%
Not stated	1	0%
**Has heart disease?**		
Yes	788	7%
No	10,996	93%
Do not know	23	0%
Refusal	9	0%
Not stated	1	0%
**Has diabetes?**		
Yes	1092	9%
No	10,707	91%
Do not know	10	0%
Refusal	7	0%
Not stated	1	0%
**Has cancer?**		
Yes	290	2%
No	11,504	97%
Do not know	11	0%
Refusal	11	0%
Not stated	1	0%
**Has osteoporosis?**		
Yes	793	7%
No	5915	50%
Valid skip	5082	43%
Do not know	16	0%
Refusal	9	0%
Not stated	2	0%
**Highest level of education**		
Less than a high school diploma or its equivalent	1934	16%
High school diploma or a high school equivalency certificate	3043	26%
Certificate/diploma – trade/college/non-university/university below Bachelor’s	3924	33%
Bachelor’s degree or university certificate/diploma/degree above Bachelor’s level	2846	24%
Not stated	70	1%
**Total household income from all sources**		
$0–$19,999	1311	11%
$20,000–$39,999	2503	21%
$40,000–$59,999	2114	19%
$60,000–$79,999	1651	14%
$80,000–$99,999	1183	11%
$100,000–$119,999	970	10%
$120,000–$139,999	617	5%
$140,000 and higher	1461	12%
Not stated	7	0%
**Immigrant status**		
Yes	2445	21%
No	9366	79%
Not stated	6	0%
**BMI, kg/m^2^**	28 ± 0.10 **^1^**	
**Physical activity level**		
<30 min/d	4168	35%
≥30 min/d but <60 min/d	7649	65%
≥60 min/d but <180 min/d	0	
≥180 min/d	0	

Abbreviations: BMI, body mass index; d, day; min, minutes; SE, standard error of the mean. ^1^ Mean ± SE.

**Table 3:** Comparison of protein intake and protein quality of diets of Canadians who consumed at least one plant food that qualified for a protein content claim in Canada, the US, or both regions.

	Non-Consumers ^1^ (n = 10,405)		Plant Protein Content Claim Consumers						*p*-Value
			Canada (n = 227)		US (n = 164)		Canada + US (n = 1021)		
	Mean	SE	Mean	SE	Mean	SE	Mean	SE	
**Total protein intake (g/d)**	79.0	0.9	91.9	5.1	85.8	15.6	82.8	2.7	0.098
Protein intake from animal foods (g/d)	52.1 ^a^	0.9	58.3 ^a^	5.3	33.7 ^b^	6.4	46.7 ^b^	2.5	0.001
Protein intake from plant foods (g/d)	26.1 ^c^	0.3	33.1 ^bc^	1.6	51.6 ^a^	10.0	35.5 ^b^	1.0	<0.001
**Total protein intake (% E)**	17.1	0.2	16.6	0.6	16.0	0.8	16.7	0.3	0.730
Protein intake from animal foods (% E)	11.4 ^a^	0.2	10.5 ^ab^	0.6	6.6 ^c^	0.7	9.5 ^bc^	0.3	0.005
Protein intake from plant foods (% E)	5.77 ^c^	0.05	6.31 ^bc^	0.48	9.61 ^a^	0.52	7.42 ^b^	0.19	<0.001
**Dietary protein quality (DIAAS) ^2^**	0.959	0.002	0.965	0.006	0.948	0.021	0.959	0.008	0.798
Protein quality from animal Foods (DIAAS) ^2^	0.990	0.001	0.987	0.006	0.992	0.025	0.989	0.008	0.800
Protein quality from plant foods (DIAAS) ^2^	0.591 ^d^	0.002	0.649 ^c^	0.015	0.766 ^a^	0.015	0.706 ^b^	0.008	<0.001
**Corrected protein intake (g/d) ^3^**	75.9	1.0	89.0	5.0	80.9	15.4	79.7	3.0	0.103
Protein intake from animal foods (g/d) ^3^	51.9 ^a^	0.9	58.2 ^a^	5.3	33.6 ^b^	6.4	46.6 ^b^	2.5	0.001
Protein intake from plant foods (g/d) ^3^	15.3 ^d^	0.2	21.5 ^c^	1.0	38.8 ^a^	7.1	25.1 ^b^	0.8	<0.001
**Corrected protein intake (% E)**	16.6	0.2	16.3	0.6	15.5	1.0	16.3	0.3	0.793
Protein intake from animal foods (% E)	11.3 ^a^	0.2	10.4 ^ab^	0.6	6.6 ^c^	0.7	9.4 ^bc^	0.3	0.004
Protein intake from plant foods (% E)	3.39 ^d^	0.03	4.09 ^c^	0.32	7.33 ^a^	0.43	5.30 ^b^	0.17	<0.001

Abbreviations: DIAAS, digestible corrected amino acid score; % E, percent energy; SE, standard error; US, United States. ANCOVA was conducted to compare protein quality and quantity among comparison groups. All models were adjusted for the following variables: misreporting status (EI/TEE), age, sex, smoking, self-perceived health, blood pressure, diabetes, heart disease, cancer, osteoporosis, education, physical activity, income, BMI, immigrant status, and weekend reference day, as previously described [24]. Post-hoc analysis with Bonferroni adjustment was used for multiple comparisons. Different superscripts within a row indicate significant differences at *p* < 0.05. ^1^ Non-consumers did not consume any food from any plant BNS food groups that qualified for a protein content claim in Canada or the United States. ^2^ DIAAS was calculated using a 0.8 true nitrogen digestibility coefficient for all foods. Median IAA for BNS food groups was applied to all foods within the BNS food group. Protein and IAA intakes across all BNS food groups were summed. The IAA requirements for children (>3 years), adolescents, and adults were used to determine the amino acid score. The lowest amino acid score was considered the DIAAS. DIAAS values were truncated at 1.0 as per recommendations for mixed diets [41]. ^3^ Corrected protein intake was obtained by multiplying the daily DIAAS by total daily protein (g) intake, the animal protein DIAAS by protein intake (g) from animal foods, or the plant protein DIAAS by protein intake (g). Different superscripts within a row indicate significant differences at *p* < 0.05.

**Table 4:** Comparison of protein intake and protein quality of diets of Canadians who consumed at least one plant food that qualified for a protein content claim in Canada, ANZ, or both regions.

	Non-Consumers ^1^ (n = 991)		Plant Protein Content Claim Consumers						*p*-Value
			Canada (n = 3)		ANZ (n = 9578)		Canada + ANZ (n = 1245)		
	Mean	SE	Mean	SE	Mean	SE	Mean	SE	
**Total protein intake (g/d)**	70.7 ^a^	2.7	74.9 ^a^	7.6	80.0 ^a^	0.9	84.6 ^a^	2.5	0.046
Protein intake from animal foods (g/d)	53.3 ^a^	2.4	57.2 ^ab^	13.4	51.6 ^ab^	0.9	49.0 ^b^	2.5	0.024
Protein intake from plant foods (g/d)	16.1 ^a^	0.7	17.7 ^bc^	5.7	27.6 ^b^	0.3	35.1 ^c^	0.9	<0.001
**Total protein intake (% E)**	18.3	0.5	15.6	2.8	16.9	0.2	16.7	0.2	0.857
Protein intake from animal foods (% E)	13.8 ^a^	0.6	12.1 ^abc^	3.3	11.0 ^b^	0.2	9.7 ^c^	0.3	<0.001
Protein intake from plant foods (% E)	4.44 ^a^	0.19	3.60 ^ab^	1.07	5.97 ^b^	0.05	7.21 ^c^	0.21	<0.001
**Dietary protein quality (DIAAS) ^2^**	0.967 ^a^	0.005	0.988 ^a^	0.010	0.958 ^a^	0.002	0.960 ^a^	0.006	0.005
Protein quality from animal foods (DIAAS) ^2^	0.977	0.010	0.999	0.006	0.991	0.001	0.988	0.006	0.487
Protein quality from plant foods (DIAAS) ^2^	0.649 ^a^	0.013	0.685 ^a^	0.022	0.589 ^b^	0.002	0.694 ^a^	0.007	<0.001
**Corrected protein intake (g/d) ^3^**	68.0	2.5	73.9	7.6	76.7	0.9	81.5	2.8	0.135
Protein intake from animal foods (g/d) ^3^	52.9 ^a^	2.4	57.1 ^a^	13.5	51.4 ^a^	0.9	48.9 ^a^	2.5	0.026
Protein intake from plant foods (g/d) ^3^	10.4 ^c^	0.5	12.0 ^ab^	3.5	16.3 ^b^	0.2	24.5 ^a^	0.7	<0.001
**Corrected protein intake (% E)**	17.8	0.6	15.5	2.7	16.4	0.2	16.3	0.3	0.638
Protein intake from animal foods (% E)	13.7 ^a^	0.6	12.1 ^abc^	3.3	11.0 ^c^	0.2	9.6 ^b^	0.3	<0.001
Protein intake from plant foods (% E)	2.90 ^c^	0.13	2.46 ^bc^	0.76	3.52 ^b^	0.03	5.07 ^a^	0.16	<0.001

Abbreviations: ANZ, Australia and New Zealand; DIAAS, digestible corrected amino acid score; % E, percent energy; SE, standard error. ANCOVA was conducted to compare protein quality and quantity among comparison groups. All models were adjusted for the following variables: misreporting status (EI/TEE), age, sex, smoking, self-perceived health, blood pressure, diabetes, heart disease, cancer, osteoporosis, education, physical activity, income, BMI, immigrant status, and weekend reference day, as previously described [24]. Post-hoc analysis with Bonferroni adjustment was used for multiple comparisons. Different superscripts within a row indicate significant differences at *p* < 0.05. ^1^ Non-consumers did not consume any food from any plant BNS food groups that qualified for a protein content claim in Canada, Australia, and New Zealand. ^2^ DIAAS was calculated using a 0.8 true nitrogen digestibility coefficient for all foods. Median IAA for BNS food groups was applied to all foods within the BNS food group. Protein and IAA intakes across all BNS food groups were summed. The Indispensable amino acid requirements for children (>3 years), adolescents, and adults were used to determine the amino acid score. The lowest amino acid score was considered the DIAAS. DIAAS values were truncated at 1.0 as per recommendations for mixed diets [41]. ^3^ Corrected protein intake was obtained by multiplying the daily DIAAS by total daily protein (g) intake, the animal protein DIAAS by protein intake (g) from animal foods, or the plant protein DIAAS by protein intake (g). Different superscripts within a row indicate significant differences at *p* < 0.05.

**Table 5:** Comparison of protein intake and protein quality of diets of Canadians who consumed at least one plant food that qualified for a protein content claim in Canada, the EU, or both regions.

	Non-Consumers ^1^ (n = 308)		Plant Protein Content Claim Consumers						*p*-Value
			Canada (n = 0)		EU (n = 10,261)		Canada + EU (n = 1248)		
	Mean	SE	Mean	SE	Mean	SE	Mean	SE	
**Total protein intake (g/d)**	71.4 ^a^	15.7			79.4 ^a^	1.0	84.6 ^a^	2.5	0.021
Protein intake from animal foods (g/d)	55.7 ^a^	12.8			51.6 ^a^	1.0	49.0 ^a^	2.5	0.028
Protein intake from plant foods (g/d)	14.7 ^c^	2.9			26.9 ^b^	0.3	35.0 ^a^	0.9	<0.001
**Total protein intake (% E)**	18.0	1.7			17.0	0.2	16.7	0.2	0.969
Protein intake from animal foods (% E)	14.5 ^a^	1.6			11.2 ^a^	0.2	9.7 ^b^	0.3	<0.001
Protein intake from plant foods (% E)	3.47 ^c^	0.25			5.90 ^b^	0.05	7.20 ^a^	0.21	<0.001
**Dietary protein quality (DIAAS) ^2^**	0.959	0.021			0.959	0.001	0.960	0.006	0.978
Protein quality from animal foods (DIAAS) ^2^	0.955	0.033			0.991	0.001	0.988	0.006	0.483
Protein quality from plant foods (DIAAS) ^2^	0.592 ^b^	0.016			0.594 ^b^	0.002	0.694 ^a^	0.007	<0.001
**Corrected protein intake (g/d) ^3^**	69.3 ^a^	15.5			76.2 ^a^	1.0	81.5 ^a^	2.8	0.015
Protein intake from animal foods (g/d) ^3^	55.3 ^a^	12.6			51.4 ^a^	1.0	48.9 ^a^	2.5	0.033
Protein intake from plant foods (g/d) ^3^	9.0 ^c^	1.7			15.9 ^b^	0.2	24.4 ^a^	0.7	<0.001
**Corrected protein intake (% E)**	17.5	1.6			16.5	0.2	16.3	0.3	0.998
Protein intake from animal foods (% E)	14.3 ^a^	1.5			11.1 ^a^	0.2	9.6 ^b^	0.3	<0.001
Protein intake from plant foods (% E)	2.09 ^c^	0.13			3.50 ^b^	0.03	5.06 ^a^	0.16	<0.001

Abbreviations: DIAAS, digestible corrected amino acid score; EU, Europe; % E, percent energy; SE, standard error. ANCOVA was conducted to compare protein quality and quantity among comparison groups. All models were adjusted for the following variables: misreporting status (EI/TEE), age, sex, smoking, self-perceived health, blood pressure, diabetes, heart disease, cancer, osteoporosis, education, physical activity, income, BMI, immigrant status, and weekend reference day, as previously described [24]. Post-hoc analysis with Bonferroni adjustment was used for multiple comparisons. Different superscripts within a row indicate significant differences at *p* < 0.05. ^1^ Non-consumers did not consume any food from any plant BNS food groups that qualified for a protein content claim in Canada or Europe. ^2^ DIAAS was calculated using a 0.8 true nitrogen digestibility coefficient for all foods. Median IAA for BNS food groups was applied to all foods within the BNS food group. Protein and IAA intakes across all BNS food groups were summed. The Indispensable amino acid requirements for children (>3 years), adolescents, and adults were used to determine the amino acid score. The lowest amino acid score was considered the DIAAS. DIAAS values were truncated at 1.0 as per recommendations for mixed diets [41]. ^3^ Corrected protein intake was obtained by multiplying the daily DIAAS by total daily protein (g) intake, the animal protein DIAAS by protein intake (g) from animal foods, or the plant protein DIAAS by protein intake (g). Different superscripts within a row indicate significant differences at *p* < 0.05.

**Table 6:** Comparison of daily nutrient intakes of Canadians who consumed at least one plant food from a BNS food group that qualified for a protein content claim in Canada, the US, or both regions.

	Non-Consumers (n = 10,405) ^1^		Plant Protein Content Claim Consumers						*p*-Value
			Canada (n = 227)		US (n = 164)		Canada + US (n = 1021)		
	Mean	SE	Mean	SE	Mean	SE	Mean	SE	
Energy (kcal)	1870.5	19.6	2206.1	127.0	1954.4	160.4	1976.1	52.9	0.233
Carbohydrates (g)	220.1 ^b^	2.4	256.6 ^ab^	15.1	274.9 ^a^	21.4	240.1 ^b^	6.2	<0.001
Carbohydrates (% E)	47.4 ^b^	0.7	46.3 ^b^	2.2	56.3 ^a^	1.4	48.5 ^b^	0.9	<0.001
Total Fibre (g/1000 kcal)	9.4 ^c^	0.1	9.9 ^bc^	1.2	15.7 ^a^	0.8	11.9 ^b^	0.3	<0.001
Total Sugars (%)	18.9	0.2	18.7	1.3	19.4	1.7	17.9	0.5	0.692
Fat (g/d)	70.0	1.6	85.2	8.1	60.2	4.0	74.5	2.8	0.265
Fat (%)	32.3	0.5	33.6	1.8	27.1	1.5	32.6	0.8	0.196
Saturated Fat (% E)	10.4 ^ac^	0.1	10.7 ^abc^	0.6	8.6 ^b^	0.6	10.4 ^ac^	0.4	0.025
Monounsaturated Fat (% E)	12.0	0.2	11.6	0.6	9.9	0.9	12.2	0.3	0.148
Polyunsaturated Fat (% E)	6.9 ^a^	0.2	8.0 ^b^	0.4	5.8 ^ab^	0.7	7.1 ^ab^	0.2	0.002
Linoleic Acid (% E)	5.9 ^a^	0.2	6.7 ^a^	0.4	4.8 ^a^	0.7	6.0 ^a^	0.2	0.009
Linolenic Acid (% E)	1.55 ^a^	0.03	2.44 ^ab^	0.36	1.62 ^ab^	0.17	1.77 ^b^	0.09	<0.001
Cholesterol (mg/1000 kcal)	149.3 ^a^	2.1	139.3 ^ab^	12.7	111.8 ^ab^	21.4	134.4 ^b^	10.7	<0.001
Alcohol (% E)	3.2 ^a^	0.3	3.5 ^ab^	0.9	0.6 ^b^	0.2	2.2 ^ab^	0.3	0.047
**Micronutrients: per 1000 kcal**									
Vitamin A (μg RAE)	365.3	11.6	428.8	126.4	389.4	74.1	373.0	17.1	0.120
Thiamin (mg)	0.86 ^a^	0.02	0.82 ^a^	0.08	0.95 ^a^	0.05	0.89 ^a^	0.04	0.019
Riboflavin (mg)	1.07 ^ab^	0.01	1.09 ^a^	0.05	1.00 ^ab^	0.10	1.01 ^b^	0.02	0.024
Niacin (mg)	21.7 ^a^	0.3	20.5 ^ab^	0.7	17.9 ^c^	0.6	19.6 ^bc^	0.6	<0.001
Vitamin B-6 (mg)	0.94	0.02	0.95	0.07	0.96	0.03	0.89	0.03	0.196
Folate (μg DFE)	116.0 ^c^	1.5	128.0 ^bc^	12.6	243.6 ^a^	14.6	147.1 ^b^	3.8	<0.001
Vitamin B-12 (mg)	2.26	0.06	2.20	0.26	1.43	0.24	2.06	0.15	0.366
Vitamin C (mg)	55.7 ^a^	1.7	61.6 ^a^	7.2	57.5 ^a^	9.2	61.2 ^a^	3.2	0.028
Vitamin D (ug)	2.6 ^ab^	0.1	2.9 ^c^	0.4	2.3 ^abc^	0.4	2.4 ^b^	0.2	0.009
Calcium (mg)	421.1 ^b^	4.1	528.0 ^a^	22.7	440.5 ^abc^	79.3	463.5 ^c^	18.7	<0.001
Iron (mg)	6.67 ^b^	0.04	6.29 ^b^	0.41	8.21 ^a^	0.33	7.41 ^a^	0.12	<0.001
Magnesium (mg)	170.5 ^b^	1.3	175.2 ^ab^	8.3	203.9 ^a^	7.5	187.6 ^a^	3.7	<0.001
Phosphorus (mg)	693.3 ^a^	4.0	741.2 ^b^	21.0	762.3 ^ab^	77.5	704.8 ^ab^	11.4	0.005
Potassium (mg)	1496.9 ^a^	12.5	1577.1 ^ab^	89.6	1667.0 ^ab^	81.2	1595.0 ^b^	28.7	<0.001
Zinc (mg)	5.59 ^b^	0.04	5.63 ^ab^	0.29	5.59 ^ab^	0.18	6.11 ^a^	0.20	0.019
Sodium (mg)	1476.4 ^a^	12.0	1457.5 ^a^	99.3	1301.8 ^a^	92.5	1537.8 ^a^	37.8	0.009

Abbreviations: BNS, Health Canada Bureau of Nutritional Sciences; DFE, dietary folate equivalent; % E, percent energy; RAE, retinol activity equivalents; SE, standard error; US, United States. ANCOVA was conducted to compare nutrient intakes among comparison groups. Post-hoc analysis with Bonferroni adjustment was used for multiple comparisons. Different superscripts within a row indicate significant differences at *p* < 0.05. ^1^ Non-consumers did not consume any food from any plant BNS food groups that qualified for a protein content claim in Canada or the United States. Different superscripts within a row indicate significant differences at *p* < 0.05.

**Table 7:** Comparison of daily nutrient intakes of Canadians who consumed at least one plant food from a BNS food group that qualified for a protein content claim in Canada, ANZ, or both regions.

	Non-Consumers (n = 991) ^1^		Plant Protein Content Claim Consumers						*p*-Value
			Canada (n = 3)		ANZ (n = 9578)		Canada + ANZ (n = 1245)		
	Mean	SE	Mean	SE	Mean	SE	Mean	SE	
Energy (kcal)	1572.3 ^c^	72.5	1945.5 ^a^	263.2	1900.6 ^b^	18.3	2022.0 ^b^	52.4	<0.001
Carbohydrate (g)	179.4 ^c^	6.4	218.1 ^a^	55.7	225.1 ^b^	2.6	243.5 ^b^	5.9	<0.001
Carbohydrates (% E)	46.7	1.3	44.0	5.2	47.7	0.6	48.1	1.1	0.098
Total Fibre (g/1000 kcal)	8.5 ^a^	0.6	5.0 ^abc^	3.5	9.6 ^b^	0.2	11.5 ^c^	0.4	<0.001
Total Sugars (% E)	21.4 ^abc^	0.9	25.7 ^b^	1.7	18.7 ^c^	0.2	18.0 ^abc^	0.6	0.017
Fat (g)	56.3 ^c^	3.3	87.3 ^a^	9.5	71.1 ^b^	1.5	76.6 ^b^	3.2	<0.001
Fat (% E)	30.6 ^b^	0.7	40.4 ^a^	2.9	32.3 ^b^	0.5	32.8 ^b^	0.9	0.013
Saturated Fat (% E)	10.0 ^b^	0.3	12.2 ^a^	0.1	10.4 ^b^	0.1	10.4 ^b^	0.4	<0.001
Monounsaturated Fat (% E)	11.6	0.3	16.0	3.1	12.0	0.2	12.0	0.3	0.452
Polyunsaturated Fat (% E)	5.9 ^b^	0.2	9.2 ^acd^	1.1	7.0 ^c^	0.2	7.3 ^d^	0.2	<0.001
Linoleic Acid (% E)	5.0 ^b^	0.2	7.4 ^acd^	1.1	5.9 ^c^	0.2	6.1 ^d^	0.2	<0.001
Linolenic Acid (% E)	1.09 ^c^	0.05	1.63 ^a^	0.36	1.60 ^b^	0.03	1.91 ^a^	0.08	<0.001
Cholesterol (mg/1000 kcal)	181.3 ^a^	10.2	147.8 ^a^	3.3	145.5 ^ab^	2.2	135.3 ^b^	8.1	<0.001
Alcohol (% E)	4.4 ^a^	0.9	0.0 ^b^	0.0	3.1 ^a^	0.3	2.5 ^ab^	0.3	<0.001
**Micronutrients: per 1000 kcal**									
Vitamin A (μg RAE)	508.8 ^a^	112.9	165.5 ^b^	55.3	352.1 ^a^	5.8	384.8 ^a^	23.8	0.002
Thiamin (mg)	0.76 ^b^	0.04	0.83 ^ab^	0.20	0.87 ^a^	0.01	0.88 ^a^	0.05	<0.001
Riboflavin (mg)	1.14 ^ab^	0.04	0.77 ^b^	0.09	1.06 ^a^	0.01	1.03 ^a^	0.02	0.013
Niacin (mg)	22.8 ^acd^	0.6	17.5 ^acd^	4.1	21.5 ^c^	0.3	19.8 ^d^	0.5	<0.001
Vitamin B-6 (mg)	1.13 ^a^	0.03	0.60 ^ab^	0.30	0.92 ^b^	0.02	0.90 ^b^	0.03	<0.001
Folate (μg DFE)	140.2 ^b^	9.2	83.5 ^b^	12.3	116.4 ^b^	1.5	143.5 ^a^	4.2	<0.001
Vitamin B-12 (mg)	2.97	0.46	1.58	0.44	2.17	0.05	2.09	0.13	0.353
Vitamin C (mg)	82.7 ^ac^	6.9	44.6 ^abc^	17.2	53.2 ^b^	1.6	61.3 ^c^	2.8	<0.001
Vitamin D (ug)	2.8	0.6	1.2	0.4	2.6	0.1	2.5	0.2	0.280
Calcium (mg)	424.9 ^b^	18.0	208.7 ^c^	48.6	421.2 ^b^	4.6	477.2 ^a^	16.1	<0.001
Iron (mg)	6.17 ^b^	0.25	4.41 ^bc^	0.84	6.75 ^c^	0.04	7.20 ^a^	0.12	<0.001
Magnesium (mg)	168.1 ^b^	6.7	81.5 ^c^	28.9	171.5 ^b^	1.2	185.4 ^a^	3.5	<0.001
Phosphorus (mg)	696.7 ^a^	17.4	545.3 ^a^	80.0	694.4 ^a^	4.4	712.5 ^a^	9.8	0.003
Potassium (mg)	1736.1 ^ac^	48.2	1092.8 ^abc^	314.7	1477.8 ^b^	12.8	1593.0 ^c^	27.4	<0.001
Zinc (mg)	5.93 ^ab^	0.22	3.99 ^b^	0.70	5.56 ^b^	0.04	6.02 ^a^	0.17	<0.001
Sodium (mg)	1495.5 ^ac^	61.0	2065.8 ^abc^	592.3	1470.9 ^c^	11.1	1520.1 ^b^	26.5	0.008

Abbreviations: ANZ, Australia and New Zealand; BNS, Health Canada Bureau of Nutritional Sciences; DFE, dietary folate equivalent; % E, percent energy; RAE, retinol activity equivalents; SE, standard error. ANCOVA was conducted to compare nutrient intakes among groups. All models were adjusted for the following variables: misreporting status (EI/TEE), age, sex, smoking, self-perceived health, blood pressure, diabetes, heart disease, cancer, osteoporosis, education, physical activity, income, BMI, immigrant status, and weekend reference day, as previously described [24]. Post-hoc analysis with Bonferroni adjustment was used for multiple comparisons. Different superscripts within a row indicate significant differences at *p* < 0.05. ^1^ Non-consumers did not consume any food from any plant BNS food groups that qualified for a protein content claim in Canada, Australia, or New Zealand. Different superscripts within a row indicate significant differences at *p* < 0.05.

**Table 8:** Comparison of daily nutrient intakes of Canadians who consumed at least one plant food from a BNS food group that qualified for a protein content claim in Canada, the EU, or both regions.

	Non-Consumers (n = 308) ^1^		Plant Protein Content Claim Consumers						*p*-Value
			Canada (n = 0)		EU (n = 10,261)		Canada + EU (n = 1248)		
	Mean	SE	Mean	SE	Mean	SE	Mean	SE	
Energy (kcal)	1651.8 ^b^	304.8			1877.6 ^a^	17.6	2021.8 ^a^	52.2	<0.001
Carbohydrates (g)	182.6 ^c^	26.4			222.1 ^b^	2.5	243.4 ^a^	5.9	<0.001
Carbohydrates (%)	46.4	3.2			47.6	0.6	48.0	1.1	0.531
Total Fibre (g/1000 kcal)	7.1 ^c^	1.7			9.6 ^b^	0.1	11.5 ^a^	0.4	<0.001
Total Sugars (% E)	22.8	3.7			18.8	0.2	18.1	0.6	0.343
Fat (g)	63.5 ^b^	13.9			69.9 ^a^	1.4	76.6 ^a^	3.2	<0.001
Fat (% E)	31.6	1.8			32.2	0.5	32.8	0.9	0.441
Saturated Fat (% E)	9.9	0.7			10.4	0.2	10.4	0.4	0.501
Monounsaturated Fat (% E)	12.7	1.1			12.0	0.2	12.1	0.3	0.896
Polyunsaturated Fat (% E)	6.2 ^ab^	0.6			6.9 ^a^	0.2	7.3 ^b^	0.2	<0.001
Linoleic Acid (% E)	5.3 ^b^	0.7			5.9 ^b^	0.2	6.1 ^a^	0.2	<0.001
Linolenic Acid (% E)	0.99 ^c^	0.11			1.57 ^b^	0.03	1.91 ^a^	0.08	<0.001
Cholesterol (mg/1000 kcal)	171.7 ^ab^	30.5			148.0 ^a^	2.4	135.3 ^b^	8.0	0.039
Alcohol (% E)	4.1	1.2			3.2	0.3	2.5	0.3	0.245
**Micronutrients: per 1000 kcal**									
Vitamin A (μg RAE)	274.1 ^b^	42.7			368.0 ^a^	11.5	384.1 ^a^	23.8	<0.001
Thiamin (mg)	0.75 ^b^	0.11			0.86 ^a^	0.01	0.88 ^a^	0.05	0.004
Riboflavin (mg)	1.09	0.12			1.06	0.01	1.03	0.02	0.298
Niacin (mg)	22.6 ^ab^	1.8			21.6 ^a^	0.3	19.8 ^b^	0.5	0.001
Vitamin B-6 (mg)	0.98	0.07			0.94	0.02	0.90	0.03	0.460
Folate (μg DFE)	84.4 ^c^	8.8			119.3 ^b^	1.5	143.3 ^a^	4.3	<0.001
Vitamin B-12 (mg)	2.53	0.45			2.24	0.06	2.09	0.13	0.531
Vitamin C (mg)	51.3 ^c^	9.3			55.9 ^b^	1.6	61.3 ^a^	2.8	<0.001
Vitamin D (ug)	2.6 ^a^	0.4			2.6 ^a^	0.1	2.5 ^a^	0.2	0.089
Calcium (mg)	376.4 ^b^	33.8			422.6 ^b^	5.5	476.4 ^a^	15.8	<0.001
Iron (mg)	5.84 ^c^	0.56			6.72 ^b^	0.04	7.19 ^a^	0.12	<0.001
Magnesium (mg)	161.1 ^b^	14.8			171.4 ^b^	1.3	185.1 ^a^	3.6	<0.001
Phosphorus (mg)	700.9 ^ab^	41.1			694.5 ^a^	4.8	712.0 ^b^	9.7	0.021
Potassium (mg)	1513.3 ^b^	81.0			1499.9 ^b^	11.8	1591.5 ^a^	27.2	<0.001
Zinc (mg)	5.80 ^ab^	0.62			5.58 ^b^	0.04	6.02 ^a^	0.17	<0.001
Sodium (mg)	1224.1 ^c^	108.9			1479.2 ^b^	11.4	1521.8 ^a^	26.3	<0.001

Abbreviations: BNS, Health Canada Bureau of Nutritional Sciences; DFE, dietary folate equivalent; EU, European Union; RAE, retinol activity equivalents; % E, percent energy; SE, standard error. ANCOVA was conducted to compare nutrient intakes among comparison groups. All models were adjusted for the following variables: misreporting status (EI/TEE), age, sex, smoking, self-perceived health, blood pressure, diabetes, heart disease, cancer, osteoporosis, education, physical activity, income, BMI, immigrant status, and weekend reference day, as previously described [24]. Post-hoc analysis with Bonferroni adjustment was used for multiple comparisons. Different superscripts within a row indicate significant differences at *p* < 0.05. ^1^ Non-consumers did not consume any food from any plant BNS food groups that qualified for a protein content claim in Canada or Europe. Different superscripts within a row indicate significant differences at *p* < 0.05.

## Data Availability

The data used in this study are available from Statistics Canada at https://www150.statcan.gc.ca/n1/en/catalogue/82M0013X (accessed on 3 July 2023). The analyzed data supporting the conclusions of this article will be made available by the authors on request.

## Supplementary Materials

The following supporting information can be downloaded at: https://www.mdpi.com/article/10.3390/nu17182987/s1. Supplementary Materials and Methods, Figure S1: Canadian regulatory framework for protein content claims using the protein efficiency ratio (PER); Figure S2: Health Canada’s interim protein content claim policy (3 December 2020–29 November 2024) on the use of the protein digestibility corrected amino acid score (PDCAAS) to be used to calculate a protein efficiency ratio (PER); Figure S3: The US regulatory framework for protein content claims using the protein digestibility corrected amino acid score (PDCAAS). The amino acid reference pattern for 2–5 year old children is used as per FAO; Figure S4: Comparison of plant BNS food groups that qualified for a “source” protein content claim in Canada and “high source” protein claim in the EU; Table S1: Identification of Health Canada’s BNS Food Group Codes that contain mainly plant derived foods with protein; Table S2: Removal and/or reclassification of food items in the Canadian Nutrient File for determining the protein quality of BNS food groups and eligibility for protein content claim under regulatory frameworks for Canada, the US, ANZ and the EU; Table S3: Specific food items that were originally allocated to 37A Legume (Non-Soy) food group and reallocated to the created BNS Food Group 37A Beans; Table S4: Specific food items that were originally allocated to 37A Legume (Non-Soy) food group and reallocated to the created BNS Food Group 37F Chickpeas; Table S5: Specific food items that were originally allocated to 37A Legume (Non-Soy) food group and reallocated to the created BNS Food Group 37G Lentils; Table S6: Specific food items that were originally allocated to 37A Legume (Non-Soy) food group and reallocated to the created BNS Food Group 37H Dried Peas; Table S7: Summary and rationale for excluding or including plant BNS food groups and/or food items from the 2018 Canadian Nutrient File for determining the protein quality of plant BNS food groups and eligibility for protein content claim under regulatory frameworks for Canada, the US, ANZ and the EU; Table S8: Assumptions and data used to calculate the protein quality and eligibility for protein content claims for plant BNS Food Groups under regulatory frameworks for Canada, the US, Australia and New Zealand and the EU; Table S9: Summary of Protein Efficiency Ratios used to calculate mean PER for plant BNS food groups under the Canadian regulatory framework for protein content claims; Table S10: Summary of true nitrogen digestibility coefficients derived from the literature and applied to plant BNS food groups for PDCAAS calculations; Table S11: Indispensable amino acid reference patterns used to determine PDCAAS of plant BNS food groups for eligibility of PCC under the US framework and DIAAS for diets; Table S12: Summary of BNS Food Groups Identified as providing plant protein, animal protein, mixture of both or negligible amounts of protein. Table S13: Comparison of daily protein intake, protein quality, and nutrient intake of Canadians who consumed at least one plant food from a BNS food group that qualified for a “Source of Protein” claim in Canada, “High Source protein” claim in Europe, or each respective claim in both regions.

## Abbreviations

ANZ	Australia and New Zealand
AAS	Amino acid score
BMI	Body mass index
BNS	Bureau of Nutritional Sciences (Health Canada)
CCHS	Canadian Community Health Survey
CFG	Canada’s food guide
CNF	Canadian nutrient file
CVD	Cardiovascular disease
DIAAS	Digestible corrected amino acid score
DFE	Dietary folate equivalent
DV	Daily value
EI	Reported energy intake
EU	European Union
IAA	Indispensable amino acids
NHANES	National Health and Nutrition Examination Survey
PCC	Protein content claim
PDCAAS	Protein digestibility corrected amino acid score
PER	Protein efficiency ratio
PQ	Protein quality
PR	Protein rating
PUMF	Public use microdata files
RA	Reference amount (Canada)
RACC	Reference amount customarily consumed (US)
RAE	Retinol activity equivalents
RDI	Reasonable daily intake (Canada)
SE	Standard error
TEE	Total energy expenditure
US	United States
% E	Percent energy
