# Supplementary material for: The Relationship Between Regulatory Frameworks for Protein Content Claims for Plant Protein Foods and the Nutrient Intakes of Canadian Adults
Source: Nutrients. 2025 Sep 18;17(18):2987. doi: 10.3390/nu17182987 (PMC12473015; doi:10.3390/nu17182987)
Supplement: Supplementary file 1 [file nutrients-17-02987-s001.zip › nutrients-3820385-supplementary.pdf]

Supplementary Materials and Methods

The relationship between regulatory frameworks for protein content claims for plant protein foods and the nutrient intakes of Canadian adults

Songhee Back [1,2], Christopher PF Marinangeli [3*], Antonio Rossi [1,2], Lamar Elfaki [4], Mavra Ahmed [1,5], Victoria Chen [1,2], Shuting Yang [1,2], Andreea Zurbau [1,2,6], Alison M Duncan [7], Cyril WC Kendall [1,2,8], John L Sievenpiper [1,2,9-11], and Laura Chiavaroli [1,2,11*]

[1] Department of Nutritional Sciences, Temerty Faculty of Medicine, University of Toronto, Toronto, Ontario, Canada
[2] Toronto 3D Knowledge Synthesis and Clinical Trials Unit and Clinical Nutrition and Risk Factor Modification Centre, St. Michael's Hospital, Toronto, Ontario, Canada
[3] Center for Regulatory Research and Innovation, Protein Industries Canada, Regina, Saskatchewan, Canada
[4] Faculty of Arts and Science, University of Toronto, Toronto, Ontario, Canada
[5] Joannah & Brian Lawson Centre for Child Nutrition, Temerty Faculty of Medicine, University of Toronto, Toronto, Ontario, Canada
[6] INQUIS Clinical Research Ltd. (formerly GI Labs), Toronto, Ontario, Canada
[7] Department of Human Health and Nutritional Sciences, University of Guelph, Guelph, ON, Canada
[8] College of Pharmacy and Nutrition, University of Saskatchewan, Saskatoon, Saskatchewan, Canada
[9] Division of Endocrinology and Metabolism, Department of Medicine, St. Michael's Hospital, Toronto, Ontario, Canada
[10] Department of Medicine, Temerty Faculty of Medicine, University of Toronto, Toronto, Ontario, Canada
[11] Li Ka Shing Knowledge Institute, St. Michael's Hospital, Toronto, Ontario, Canada

Supplemental Materials and Methods

2.1. Data Sources

2.1.1. The 2015 Canadian Community Health Survey-Nutrition (CCHS)

The CCHS is a cross-sectional, voluntary, national health survey conducted by Statistics Canada [1]. CCHS is a representative sample of the population across 10 Canadian provinces. Individuals who were full-time members of the Canadian Forces, living in Canada's two territories, on reserves, in Aboriginal settlements, or in institutions were excluded. A complete description of the sampling method used for the CCHS is provided elsewhere [1]. Nutrition Public Use Microdata Files (PUMF) from the CCHS were used to quantify food intakes. This is the first open-access PUMF released for CCHS. It contained all the information required for this study, such as demographics, dietary patterns (including details on what foods were eaten and quantities), and health information; including self-reported physical activity, height and weight, and health conditions [2].

A detailed overview of the CCHS is provided elsewhere [1]. Briefly, dietary data were collected using the United States Department of Agriculture's five-step automated multi-pass method, adapted and modified for the Canadian population to conduct computer-assisted interviews [3]. Survey interviews were conducted between January 2, 2015, and December 31, 2015, and consisted of two separate questionnaires per household: 1) a 24-hour dietary recall to assess all food and beverage intake over 24 hours, and 2) a general health questionnaire to collect sociodemographic, anthropometric and health status data. Overall, 20,487 individuals completed the initial 24-hour dietary recall. A random sub-sample (n=7,608) completed the second recall by phone 3–10 days after. Response rates were 61.6% and 68.6%, respectively for the initial and second recall. For the present analysis, the first 24-hour dietary recall from the 2015 CCHS-Nutrition PUMF data was used [2].

2.1.2. Inclusion and Exclusion Criteria for Data Collection

Sampling criteria for this study are summarized in **Figure 1**. Participants with invalid dietary recalls or reporting no food or energy intake were excluded (n=11).

As misreporting was used as a covariate in all analyses, those identified as respondents who lacked sufficient information to adjust for misreporting status were also excluded (n=2,998). Misreporting status was based on previously published methods that compared reported energy intake (EI) and total energy expenditure (TEE) (i.e., overreporting and underreporting of EI) [4]. The TEE of respondents was predicted based on the variables age, sex, height, weight, and physical activity levels using the Institute of Medicine's equations [5]. Body Mass Index (BMI) was calculated using measured height and weight for the respondents. When not measured, a correction factor was applied to the adults with self-reported height and weight, and sex-adjusted [6]. Cut-offs to define sedentary, low active, active and very active for physical activity levels were applied based on Health Canada's Guidance [1]. Underreporting and over-reporting were defined as the ratio of EI/TEE and used as a misreporting variable to determine the misreporting status of dietary recalls. Therefore, under-reporting and over-reporting were defined as the ratio of EI/TEE < 0.7 and >1.42, respectively, whereas those in between were considered plausible reporters [4]. Given that there was no TEE equation for underweight subjects, all individuals with a BMI <18.5 (n=377) were excluded.

All individuals <19 years (n=5,493) and participants who were breastfeeding (n=168) were also excluded [20, 25]. Pregnant individuals were not identified in the survey. After exclusions, the final sample size was 11,817 adults (≥19 years) which was pooled data from male (n= 5670) and female (n = 6147) sex groups.

2.1.3. The Canadian Nutrient File and Bureau of Nutritional Science Food Groups

For the nutritional analysis of foods, the CCHS was coded by Health Canada using the 2015 Canadian Nutrient File (CNF) and Health Canada Bureau of Nutritional Science (BNS) food groups [1,8]. The CNF is a national food and nutrient database that reflects the nutrient composition of foods in Canada. Since the completion of the CCHS, the CNF has undergone some updates. For this study, the 2018 CNF was downloaded on July 23, 2019, and included macronutrient and micronutrient composition data of 5,596 foods. Each food from the CNF is classified under a BNS food group, then subclassified under a subgroup. For example, foods allocated to the "Milks" group (BNS food group code: 10) are further categorized into an appropriate BNS subgroup "10A-K". BNS food groups that were primarily comprised of plant foods were identified (**Supplemental Table S1).**

To more accurately aggregate the protein and IAA levels of plant BNS food groups, a number of changes to the BNS food group structure were made for this study (**Supplemental Table S2).** Briefly, as previously reported [9], 94 foods were missing in the 2018 CNF compared to the 2015 CNF. These missing food items were identified and assigned to the appropriate BNS food group. Foods that had missing protein and/or IAA data were also removed (n= 1,921) [9]. Sweet potatoes and yams were reassigned from group 36P (cucumber, immature beans, brussel sprouts, beets, turnips) to the "Potato" group (39A) to more accurately reflect how these foods are consumed [9].

Additional changes to the database were made for this study that were distinct from previous analyses. Granola bars were re-classified from BNS food group 07A (cookies) to a newly formed group 07C (granola bars). The plant-based beverage group (BNS 10J) was re-classified into two separate groups, soy-based beverages (BNS 10J) and almond-based beverages (BNS 10L), to account for their substantial differences in protein content. The legume group (37) was further segregated to better reflect differences in PQ and nutrient composition. The new food subgroups under the Legume group (37) were dry beans (37A), legumes (soy-based) (37B), sprouted legumes (non-soy) (37C), sprouted legumes (soy) (37D), and meat alternatives (unidentified protein source) (37E), chickpeas (37F), lentils (37G), and dried peas (37H) (**Supplemental Tables S3-S6**). Additionally, five foods were reassigned to new food groups due to misclassification and better alignment to how they are consumed in dietary patterns (**Supplemental Table S2,** items 7-9).

In preparation for determining whether plant BNS food groups were eligible for a PCC across regions (Section 2.2), additional food exclusions were made to prevent redundancies, account for cooked vs. raw composition, and overweighting of similar foods within plant BNS food groups (**Supplemental Table S7).** Briefly, food items that contained animal protein, combination dishes, and isolates and concentrates were removed from the data set. Plant BNS food groups considered to be a negligible source of protein were excluded from PQ and PCC assessments across plant BNS food groups. Some food within plant BNS food groups (i.e. legumes 37A dry beans) had protein and IAA composition data for foods with an unsuitable method of preparation for consumption (i.e. uncooked dry beans); and were removed from the aggregated analysis. However, if a cooked method of preparation was not present, the food remained in the analysis. Similarly, food items prepared differently than what was outlined in Health Canada's *Table of Reference Amounts* were also removed. Some food items were present in the CNF with varying methods of preparation. The protein and IAA composition of these items were averaged within the BNS food group, then combined with other compositional data in the same group to derive median protein and IAA levels. These assumptions prevented duplication of food items and helped mitigate the protein and IAA composition of plant BNS food groups to be falsely skewed for protein content, PQ and eligibility for a PCC. Plant foods that contained animal fats were included in the protein and IAA assessments. Finally, if a BNS food group only contained one specific type of food, all methods of preparation were included.

2.2. PCC Validation for BNS Food Groups for Each Region

A stepwise summary of the methods across sections 2.2-2.4 is provided in **Figure 2**.

2.2.1. Summary of PCC Regulatory Frameworks for Canada, the US, ANZ, and the EU.

Canada's PCC regulatory framework was compared with those of the US, ANZ, and the EU. These PCC frameworks were chosen because these regions are governed by like-minded regulatory agencies [10,11] and have similar food systems compared to Canada. PCC frameworks are summarized in **Table 1**, where each region permits two levels of PCCs: "source" and "high source". Detailed explanations for calculating the PCC of these four regulatory frameworks have been previously described [12] and are briefly summarized below.

Methods for assessing the PQ for eligibilty for a PCC in Canada and the US are summarized in **Supplemental Figures S1-S3**. For Canada, the PER rat growth bioassay involves feeding growing weanling rats either the test protein or a casein (control) as the source of protein [19] **(Supplemental Figure S1)**. The ratio of weight gain per gram protein intake is assessed for each group. The test (weight gain/g protein intake):control (weight gain/g protein intake) ratio is calculated resulting in an unadjusted PER. To account for intralaboratory variation, a 2.5 correction factor, corresponding to the PER for casein is applied providing an adjusted PER. The protein rating of a food is determined by multiplying the adjusted PER by the protein content in a Reasonable Daily Intake (RDI) of a specific food. The RDI is a regulated level of intake corresponding to the amount of a food typically consumed in a single day in Canada [20]. When an RDI for a food does not exist, the amount of protein in a reference amount (RA) of the food can be used [21]. The RA is a regulated serving size of a food in Canada based on historical consumption [22]. In Canada, using the PER method, a protein rating of ≥20 qualifies for a "good source", and ≥40 qualifies for an "excellent source" of protein claim [13].

In December 2020, given the ongoing logistical challenges with the utilization of the PER, the Government of Canada provided industry with an interim policy for generating a PER from a known PDCAAS for a food to support a PCC [23-26] **(Supplemental Figure S2)**. From this interim policy, a PER is generated by multiplying a PDCAAS for a food by 2.5, which assumes that a PDCAAS of 1.0 demonstrates equivalency to casein, a high quality protein [12,14,23,25]. It is important to note that the relationship between in vivo PER determination and derivation of PER from PDCAAS has not been scientifically validated [12].

In the US, the PDCAAS of a food is calculated according to methods outlined by the FAO's 1991 report [15] using true N digestibility coefficients and IAA (mg/g protein) of the food [27] **(Supplemental Figure S3)**. An amino acid score (AAS) is calculated by dividing the level of each IAA (mg/g protein) in the food by the corresponding IAA (mg/g protein) requirements from the reference pattern for children 2-5 years of age. This corresponds to the methods from the FAO's 1991 report [27]. The amino acid with the lowest score is multiplied by the digestibility coefficient to give a PDCAAS. For foods with multiple sources of protein, a weighted true N digestiblity coefficient can be applied [27]. The PDCAAS is multiplied by the level of protein per Reasonable Amount Customarily Consumed (RACC) of a food to provide a corrected level of protein. Similar to the RA in Canada, a RACC is a regulated serving size. A PDCAAS of 1.0 indicates that all IAA requirements (mg/g protein) are being met by the food. All PDCAAS values >1.00 are truncated to 1.00 [27]. When the corrected level of protein is ≥10% and ≥20% of

the daily value for protein (50 g/day) [15], the food can qualify for a "good" or "excellent" source of protein, respectively [16].

PCCs in ANZ and the EU are based on protein quantity or energy from protein, per serving respectively. ANZ assesses the amount of protein in grams per serving, with ≥5 g per serving required for a "general source" and ≥10 g per serving for a "good source" of protein claim [17]. The EU measures the percentage of energy from protein where ≥12% energy per serving is a "source" and ≥20% energy per serving is a "high source" of protein [18].

2.2.2. Assessment of Plant BNS Food Groups for a PCC under Canadian, US, ANZ, and EU Regulations

Assumptions and data used to calculate the PQ and/or eligibility for a PCC for plant BNS food groups are summarized in **Supplemental Table S8.**

2.2.2.1. Eligibility of BNS Food Groups for a PCC under Canadian Regulations

To assess the eligibility of a BNS food group for a PCC under Canadian regulations, Canada's PER PCC regulations were used alongside the interim PDCAAS policy in place at the time of this analysis as described previously (Section 2.2.1; **Supplemental Figures S1-S2**). For each plant BNS food group, the median protein level across foods was determined. For each food within each BNS food group, RDIs were allocated based on Schedule K of Canada's Food and Drug Regulations [28]. When an RDI was not available, the RA was used [14]. The average RDI and RA for food items within the BNS food group were determined. The protein rating was calculated by multiplying the PER by the average RDI or RA as per Canadian requirements [21].

If PER data were available, the PER method was applied to the BNS subgroup to determine eligibility for a PCC under Canadian regulations. PER values from the Canadian Food Inspection Agency were applied [14] **(Supplemental Table S9)**. When BNS food groups contained different foods with different PERs, the mean PER was used.

When a PER was not available for a plant BNS food group, a PER was calculated based on Health Canada's interim policy of deriving a PER from a PDCAAS. Briefly, median levels of protein and each IAA (mg/g protein) within each plant BNS group were determined. The median level of each IAA was divided by the IAA requirement (mg/g protein) for 2-5 year old children as per the FAO's recommendation **(Supplemental Table S11)** [27]. The lowest ratio was determined to be the AAS. The peer-reviewed literature was used to determine the true N digestibility coefficients of plant foods to be applied to PDCAAS assessments. When >2 true N digestibility coefficients were available for a plant BNS food group, the mean true N coefficient was used to calculate the PDCAAS for the food group **(Supplemental Table S10)**. When ≤2 true N digestibility coefficients were available or not available for a plant BNS food group, as was the case for fruit and vegetable groups, a conservative 0.8 N digestiblity coefficient was applied to the entire BNS food group based on previous analyses [9]. The PDCAAS for the BNS food group was calculated by multiplying the AAS by the N digestibility coefficient. The PER for each BNS food group was calculated by multiplying PDCAAS by 2.5 as outlined in Section 2.2.1. Once a PER was generated, a protein rating was calculated to determine the eligibility of each plant BNS food group for a PCC.

2.2.2.2. Eligibility of BNS Food Groups for a PCC under US, ANZ and EU Regulations

For the US, PDCAAS for each BNS food group was determined as previously described (Section 2.2.1) and used the same true N digestibility assumptions used in applying PDCAAS under the Canadian framework (Section 2.2.2.1). Corrected protein was calculated by multiplying PDCAAS by the amount of

protein per Canadian RA. The RDI was not used. The amount of corrected protein was divided by an assumed DV of 50 g/day as per **Supplemental Figure S3**.

For the ANZ PCC framework, the median total protein (g/100g serving) was multiplied by the average RA for each plant BNS food group. Eligibility for a PCC in the EU was determined by evaluating the median protein per 100 g in the plant BNS food group. This was multiplied by the Atwater conversion factor for protein (4 kcal/g protein) and divided by the median energy per 100 g across food items in the same group.

For all calculations, identification of a BNS food group that qualified for a PCC was based on requirements for a "source of protein" claim (**Table 1**). However, "high source" PCC frameworks were used in a sensitivity analysis under the EU regulations.

2.3. Identification of Consumers and Non-Consumers of "Source of Protein" Plant Foods

Respondents from the CCHS of 2015 were characterized as "consumers" if they consumed ≥1 plant food, regardless of amount, from a plant BNS food group characterized as a "source of protein" from PCC regulations from Canada, the US, ANZ, or the EU. This study assumed that the presence of a PCC facilitated the purchase and consumption of a plant food qualifying for a PCC under a regulatory framework. Respondents who did not consume any plant food from a plant BNS food group eligible in a PCC framework were characterized as a "non-consumer."

2.4. Calculating the DIAAS and the Corrected Protein Intakes in PCC consumers and non-consumers.

The PQ of the diets for each CCHS participant was calculated and accounted for protein complementarity from different food sources using the Digestible Indispensable Amino Acid Score (DIAAS). Consumption of protein from all BNS food groups and food items was used to determine the DIAAS of daily diets for each individual. Published in 2013 by the FAO, DIAAS utilizes updated IAA reference patterns across the lifespan (mg/g protein) and uses ileal IAA digestibility coefficients [29]. However, if an ileal digestibility coefficient is not available, true N protein digestibility is to be used [29]. This was the case for this study. A conservative true N digestibility coefficient of 0.8 was applied to all BNS food groups, based on previous analyses [9].

Briefly, for each individual, 24-hour recall data were used to estimate the protein intake from each BNS food group. Protein from each BNS food group was derived from compositional data of food items from the CNF. IAA estimates for plant and animal BNS foods groups were calculated as median values across food items within the group. Daily IAA (mg/g protein) intakes from each BNS food group were summed and each was divided by the corresponding IAA (mg/g protein) recommendation from the IAA reference scoring for older children, adolescents, and adults [29] (**Supplemental Table S11**). This was the appropriate reference pattern given that adults ≥19 years of age were used in this analysis. The IAA score was the IAA with the lowest ratio. To determine the DIAAS of diet, the IAA score was multiplied by the 0.8 true N digestibility coefficient. A DIAAS of 1.00 represents a dietary pattern meeting 100% IAA requirements. As per the standard method for calculating the DIAAS for mixed diets, DIAAS values >1.00 were truncated to 1.00 [29].

Corrected daily protein intakes (g/day) were determined by multiplying the daily DIAAS for each individual by their total crude protein intake (g/day) based on specific foods consumed. To understand the differences in PQ and corrected protein, a DIAAS for plant and animal sources was also calculated.

2.5. Macronutrient and Micronutrient Intakes of Participants in each PCC group

Although BNS food codes were used to determine allocation to PCC groups and PQ of daily diets, all macronutrient and micronutrient intakes were based on the composition of specific foods consumed by each participant.

2.6. Statistical Analysis

Data cleaning and preparation were completed using R Studio version 2024.04.2+764, and all statistical analyses were conducted using SAS version 9.4 (SAS Institute Inc., Cary, N.C., USA). To ensure national representation, estimates were weighted using the survey weights provided by Statistics Canada. Variance estimation was performed using the bootstrap balanced repeated (BRR) replication with 500 replicates. All nutrients were expressed as either a percentage of energy (%E) or per 1000 kcal to adjust for energy. To satisfy the normality requirement of statistical tests, the values of the nutrients were transformed to approximate a normal distribution using the Box-Cox method.

Participant characteristics are described using mean and standard error (SE) for continuous normally distributed variables and number and frequency for categorical variables.

Three separate analyses were used to compare PQ, protein quantity, and nutrient intakes (macronutrient and micronutrients) between consumers and non-consumers of plant protein foods meeting a PCC in Canada compared to either the US, ANZ, or the EU. Participants in each of the three analyses were classified as meeting the PCC in one of the comparison frameworks, both comparison frameworks, or neither of the comparison frameworks (non-consumers). Comparisons were assessed using ANCOVA with post-hoc Bonferroni adjustment for multiple comparisons (PROC SURVEYREG). All models were adjusted for the following variables: misreporting status (EI/TEE), age, sex, smoking, self-perceived health, blood pressure, diabetes, heart disease, cancer, osteoporosis, education, physical activity, income, BMI, immigrant status, and weekend reference day, as previously described [7]. Two-tailed p-values at <0.05 were considered statistically significant.

Given that the EU's PCC framework is based on energy, substantially more plant BNS food groups qualified for a "source of" PCC compared to Canada, ANZ, and the US. A sensitivity analysis was completed to compare the PQ, crude and corrected protein quantity, and nutrient intakes (macro and micronutrients) between consumers of plant foods eligible for a "high source" PCC according to the EU, "source of protein" PCC in Canada, and non-consumers. The sensitivity analysis was limited to the EU framework because the higher threshold for "high source of protein" claims in the US and ANZ limited the number of plant BNS food groups that met the PCC requirements.

References

1. Health Canada. Reference Guide to Understanding and Using the Data. 2015 Canadian Community Health Survey—Nutrition. Availabe online: https://www.canada.ca/content/dam/hc-sc/documents/services/food-nutrition/food-nutrition-surveillance/ReferenceGuide2015CCHS-Nutr_Eng_Final_06192017.pdf (accessed on February 7, 2025).

2. Statistics Canada. Canadian Community Health Survey—Nutrition: Public Use Microdata File, 2015. Canada, S., Ed. Ottawa, ON, Canada, 2018.

3. United States Department of Agriculture. AMPM—USDA Automated Multiple-Pass Method. Agriculture, U.S.D.o., Ed. Washington, DC, USA, 2019.

4. Garriguet, D. Accounting for misreporting when comparing energy intake across time in Canada. *Health Rep* **2018**, *29*, 3-12.

5. Institute of Medicine Food and Nutrition Board. Dietary Reference Intakes for Energy, Carbohydrate, Fiber, Fat, Fatty Acids, Cholesterol, Protein, and Amino Acids (Macronutrients). Press, N.A., Ed. Washington, DC, USA, 2005.

6. Shields, M.; Gorber, S.C.; Janssen, I.; Tremblay, M.S. The bias in self-reported estimates of obesity in Canadian health surveys: An update on establishing correction equations for adults. *Canadian Journal of Diabetes* **2011**, *35*, 200, doi:10.1016/S1499-2671(11)52226-9.

7. Wang, Y.F.; Chiavaroli, L.; Roke, K.; DiAngelo, C.; Marsden, S.; Sievenpiper, J. Canadian Adults with Moderate Intakes of Total Sugars have Greater Intakes of Fibre and Key Micronutrients: Results from the Canadian Community Health Survey 2015 Public Use Microdata File. *Nutrients* **2020**, *12*, doi:10.3390/nu12041124.

8. Health Canada. Canadian Nutrient File—About Us. Availabe online: https://www.canada.ca/en/health-canada/services/food-nutrition/healthy-eating/nutrient-data/canadian-nutrient-file-compilation-canadian-food-composition-data-users-guide.html (accessed on February 11, 2025).

9. Marinangeli, C.P.F.; Fabek, H.; Ahmed, M.; Sanchez-Hernandez, D.; Foisy, S.; House, J.D. The effect of increasing intakes of plant protein on the protein quality of Canadian diets. *Applied Physiology, Nutrition, and Metabolism* **2021**, *46*, 771-780, doi:10.1139/apnm-2020-1027 %M 33591857.

10. Health Canada. Regulatory modernization of foods for special dietary use and infant foods: Divisions 24 and 25 of the Food and Drug Regulations. Section 2.3 International context. Availabe online: https://www.canada.ca/en/health-canada/programs/consultation-regulatory-modernization-foods-special-dietary-use-infant-foods/document.html#a2.3 (accessed on February 7, 2025).

11. Government of Canada. Canada – United States of America Food Safety Systems Recognition Arrangement. Availabe online: https://inspection.canada.ca/en/importing-food-plants-animals/food-imports/foreign-systems/recognition-arrangement (accessed on February 7, 2025).

12. Marinangeli, C.P.F.; Foisy, S.; Shoveller, A.K.; Porter, C.; Musa-Veloso, K.; Sievenpiper, J.L.; Jenkins, D.J.A. An Appetite for Modernizing the Regulatory Framework for Protein Content Claims in Canada. *Nutrients* **2017**, *9*, 921.

13. Health Canada. Table of Chemical, Physical and Nutritional Characteristics of Food: Item 44 and 45 Availabe online: https://www.canada.ca/en/health-canada/services/food-nutrition/legislation-guidelines/acts-regulations/incorporation-reference/table-chemical-physical-nutritional-characteristics-food.html (accessed on February 10, 2025).

14. Canadian Food Inspection Agency. Elements within the Nutrition Facts table: Protein. Availabe online: https://inspection.canada.ca/en/food-labels/labelling/industry/nutrition-labelling/elements-within-nutrition-facts-table#c7 (accessed on February 10, 2025).

15. Food and Drug Administration; Department of Health and Human Services. Nutrition labeling of food. 21 CFR 101.9(b)(7). Availabe online: https://www.ecfr.gov/current/title-21/part-101/section-101.9#p-101.9(c)(7) (accessed on February 5, 2025).

16. Food and Drug Administration; Department of Health and Human Services. Nutrient content claims for "good source," "high," "more," and "high potency.. 21 CFR 101.54. Availabe online: https://www.ecfr.gov/current/title-21/section-101.54 (accessed on February 10, 2025).

17. Food Standards Australia New Zealand. Schedule 4 – Nutrition, health and related claims: F2024C01127 (C06). Availabe online: https://www.legislation.gov.au/F2015L00474/latest/text (accessed on February 5, 2024).

18. European Union. Regulation (EC) No 1924/2006 of the European Parlimaent and of the council of of 20 December 2006 on nutrition and health claims made on foods (EUR-Lex - 02006R1924-20141213). Availabe online: http://data.europa.eu/eli/reg/2006/1924/2014-12-13 (accessed on February 5, 2024).

19. Health Canada. Method FO-1: Determination of protein rating. Availabe online: https://www.canada.ca/content/dam/hc-sc/documents/services/food-nutrition/research-programs-analytical-methods/analytical-methods/chemical-compendium-analysis-foods/determination-protein-rating/determination-protein-rating.pdf (accessed on February 4, 2025).

20. Canadian Food Inspection Agency. Daily Intake: Reasonable Daily Intake (Schedule K). Availabe online: https://inspection.canada.ca/en/food-labels/labelling/industry/nutrition-labelling/nutrition-facts-table#s14c6 (accessed on February 10, 2025).

21. Canadian Food Inspection Agency. Specific nutrient content claim requirements: Protein claims. Availabe online: https://inspection.canada.ca/en/food-labels/labelling/industry/nutrient-content/specific-requirements#a3 (accessed on July 14, 2025).

22. Government of Canada. Nutrition labelling - Table of reference amounts for food. Availabe online: https://www.canada.ca/en/health-canada/services/technical-documents-labelling-requirements/nutrition-labelling-table-reference-amounts-food.html#c (accessed on October).

23. Health Canada. Measuring the protein quality of foods. Availabe online: https://www.canada.ca/en/health-canada/services/food-nutrition/legislation-guidelines/policies/measuring-protein-quality-foods.html (accessed on February 11, 2025).

24. Marinangeli, C.P.F.; House, J.D. Potential impact of the digestible indispensable amino acid score as a measure of protein quality on dietary regulations and health. *Nutr Rev* **2017**, *75*, 658-667, doi:10.1093/nutrit/nux025.

25. Government of Canada. Canada Gazette, Part I, Volume 157,Number 44: Regulations Amending Certain Regulations Concerning Food Additives and Compositional Standards, Microbiological Criteria and Methods ofAnalysis for Food. Section 4. Lack of flexibility for measuring the protein quality of foods. Availabe online: https://canadagazette.gc.ca/rp-pr/p1/2023/2023-11-04/html/reg2-eng.html (accessed on February 10, 2025).

26. Government of Canada. Canada Gazette, Part II, Volume 158. Number 26. Regulations Amending Certain Regulations Concerning Food Additives and Compositional Standards, Microbiological Criteria and Methods ofAnalysis for Food: SOR/2024-244. Availabe online: https://www.gazette.gc.ca/rp-pr/p2/2024/2024-12-18/html/sor-dors244-eng.html (accessed on February 10, 2025).

27. FAO; WHO. *Protein quality evaluation: Report of the Joint FAO/WHO Expert Consultation, FAO Food and Nutrition: Paper 51*; 0254-4725; Food and Agriculture Organization of the United Nations and The World Health Organization: Rome, Italy, 1991; pp 1-66.

28. Government of Canada. Food and Drug Regulations. Schedule K: Reasonable Daily Intake for Various Foods. Availabe online: https://laws.justice.gc.ca/eng/regulations/c.r.c.,_c._870/page-105.html#h-580603 (accessed on February 11, 2025).

29. FAO. *Dietary protein quality evaluation in human nutrition: Paper 92*; Food and Agriculture Organization of the United Nations and The World Health Organization: Rome, Italy, 2013.

Supplemental Figures

The relationship between regulatory frameworks for protein content claims for plant protein foods and the nutrient intakes of Canadian adults

Songhee Back [1,2], Christopher PF Marinangeli [3]*, Antonio Rossi [1,2], Lamar Elfaki [4], Mavra Ahmed [1,5], Victoria Chen [1,2], Shuting Yang [1,2], Andreea Zurbau [1,2,6], Alison M Duncan [7], Cyril WC Kendall [1,2,8], John L Sievenpiper [1,2,9-11], and Laura Chiavaroli [1,2,11]*

[1] Department of Nutritional Sciences, Temerty Faculty of Medicine, University of Toronto, Toronto, Ontario, Canada
[2] Toronto 3D Knowledge Synthesis and Clinical Trials Unit and Clinical Nutrition and Risk Factor Modification Centre, St. Michael's Hospital, Toronto, Ontario, Canada
[3] Center for Regulatory Research and Innovation, Protein Industries Canada, Regina, Saskatchewan, Canada
[4] Faculty of Arts and Science, University of Toronto, Toronto, Ontario, Canada
[5] Joannah & Brian Lawson Centre for Child Nutrition, Temerty Faculty of Medicine, University of Toronto, Toronto, Ontario, Canada
[6] INQUIS Clinical Research Ltd. (formerly GI Labs), Toronto, Ontario, Canada
[7] Department of Human Health and Nutritional Sciences, University of Guelph, Guelph, ON, Canada
[8] College of Pharmacy and Nutrition, University of Saskatchewan, Saskatoon, Saskatchewan, Canada
[9] Division of Endocrinology and Metabolism, Department of Medicine, St. Michael's Hospital, Toronto, Ontario, Canada
[10] Department of Medicine, Temerty Faculty of Medicine, University of Toronto, Toronto, Ontario, Canada
[11] Li Ka Shing Knowledge Institute, St. Michael's Hospital, Toronto, Ontario, Canada

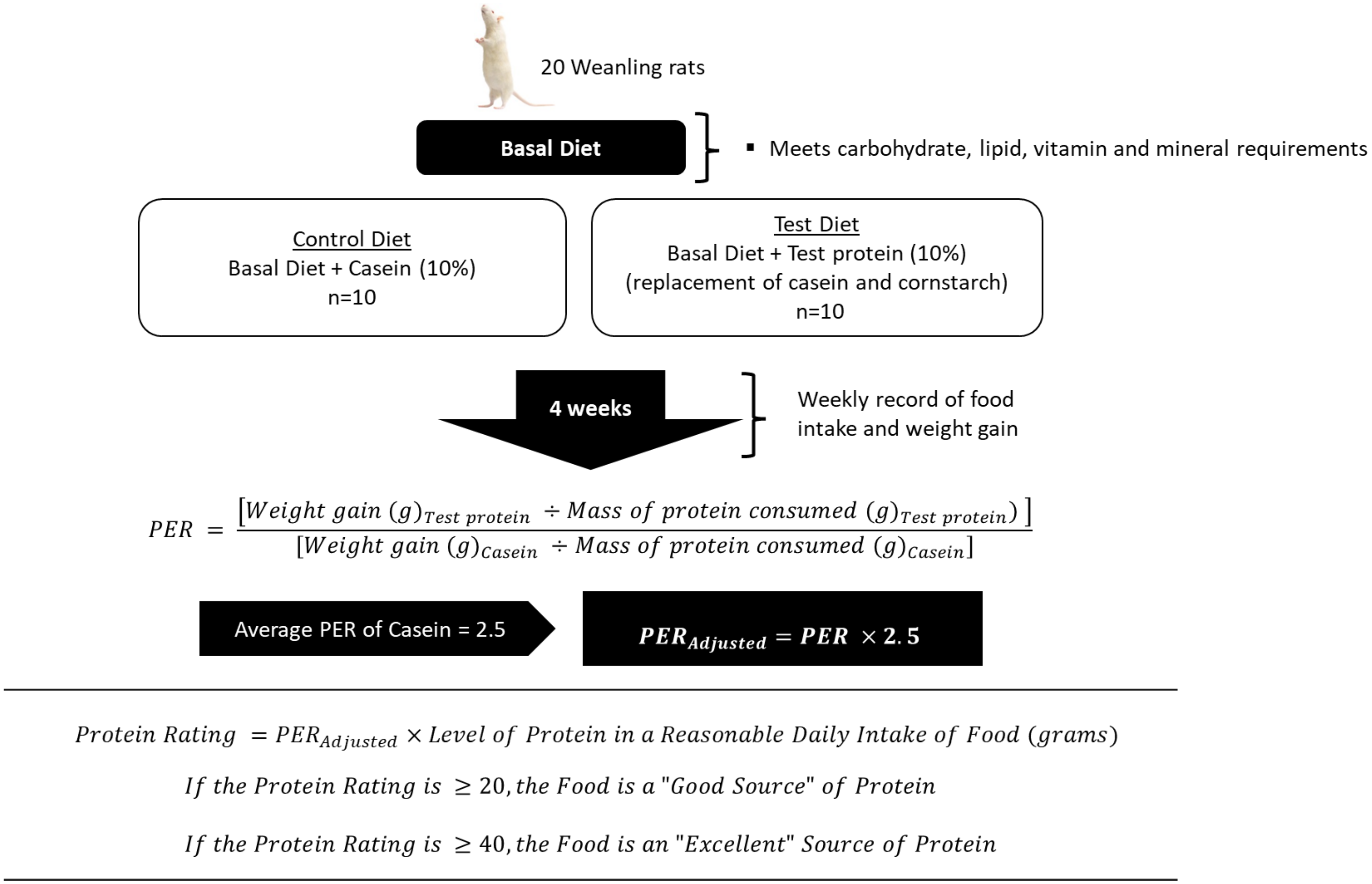

Figure S1. Canadian regulatory framework for protein content claims using the protein efficiency ratio (PER). Adapted from Marinangeli and House [1].

PDCAAS for a food x 2.5 = Estimated PER for a Food

Protein Rating = estimated PER for a food x grams of Protein in a Reasonable Daily Intake

Figure S2. Health Canada's interim protein content claim policy (December 3, 2020 – November 29, 2024) on the use of the protein digestibility corrected amino acid score (PDCAAS) to be used to calculate a protein efficiency ratio (PER). Adapted from Marinangeli and House [1].

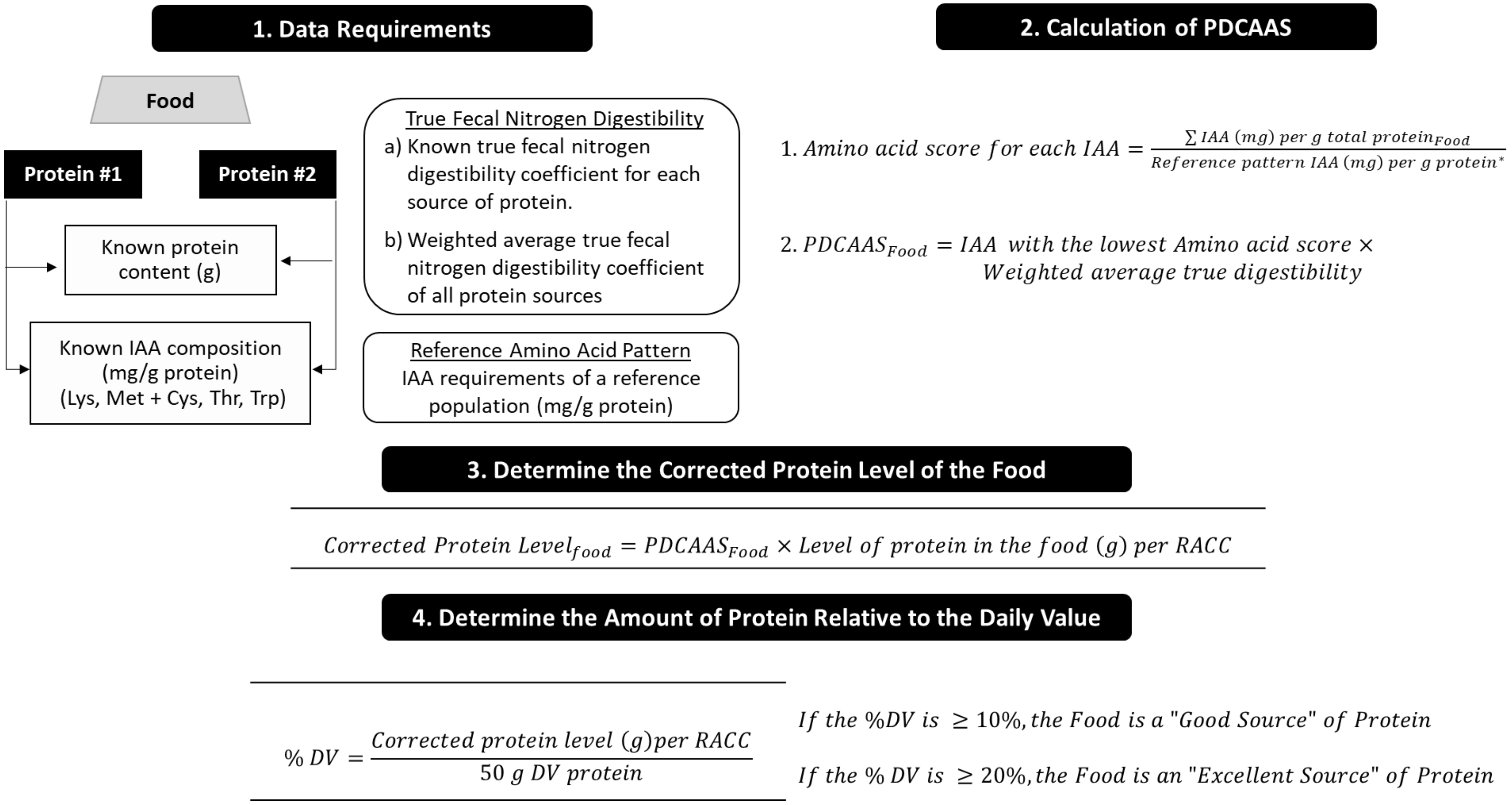

Figure S3. The US regulatory framework for protein content claims using the protein digestibility corrected amino acid score (PDCAAS). The amino acid reference pattern for 2-5year old children is used as per the FAO [2]. RAAC, reference amount customarily consumed. Adapted from Marinangeli and House [1].

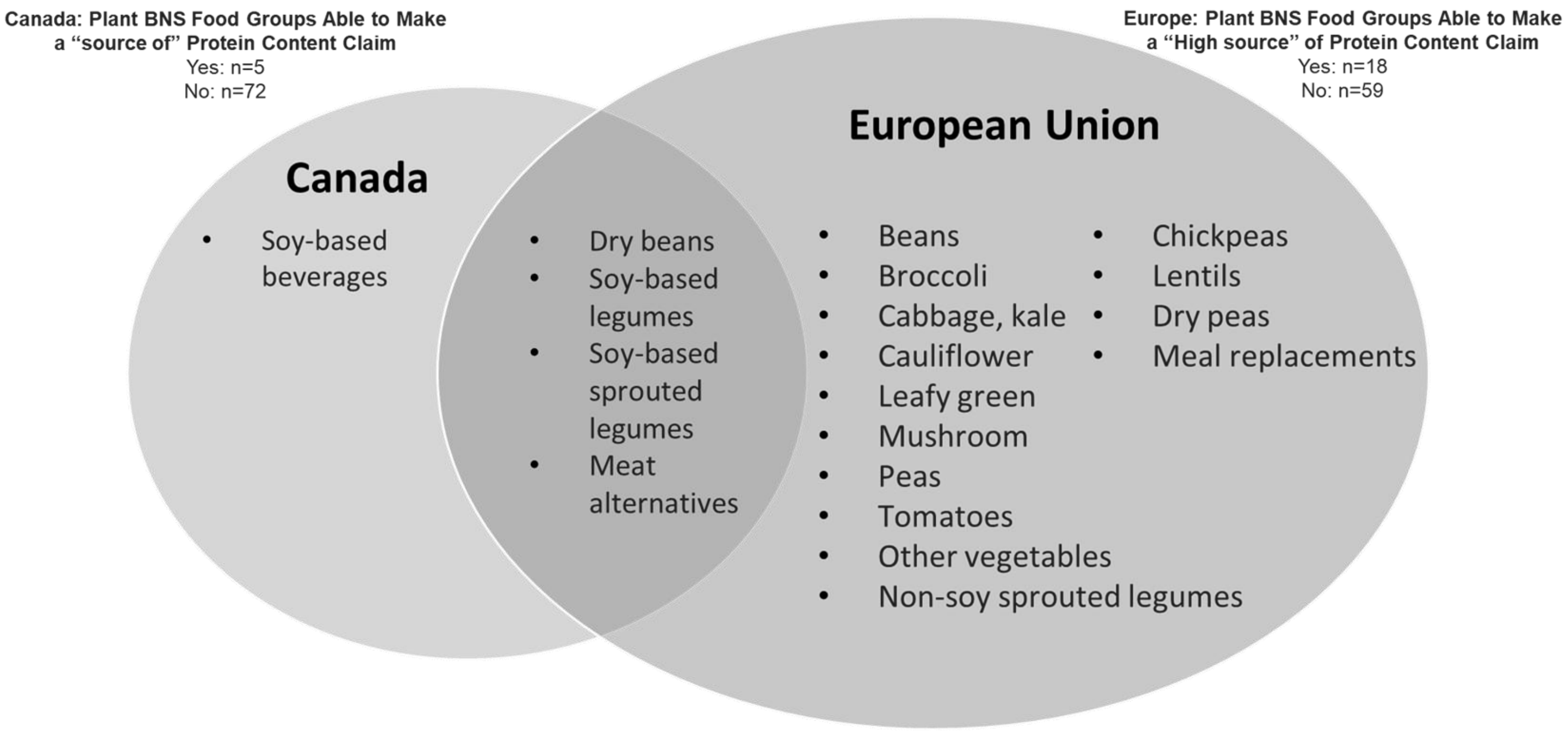

Supplemental Figure S4. Comparison of plant BNS food groups that qualified for a "source" of protein content claim in Canada and "high source" of protein content claim in the EU. Areas of overlap represent plant BNS food groups that qualified for a PCC using regulatory frameworks in both regions.

References

1. Marinangeli, C.P.F.; House, J.D. Potential impact of the digestible indispensable amino acid score as a measure of protein quality on dietary regulations and health. *Nutr Rev* **2017**, *75*, 658-667, doi:10.1093/nutrit/nux025.

2. FAO; WHO. *Protein quality evaluation: Report of the Joint FAO/WHO Expert Consultation, FAO Food and Nutrition: Paper 51*; 0254-4725; Food and Agriculture Organization of the United Nations and The World Health Organization: Rome, Italy, 1991; pp 1-66.

Supplemental Tables

The relationship between regulatory frameworks for protein content claims for plant protein foods and the nutrient intakes of Canadian adults

Songhee Back [1,2], Christopher PF Marinangeli [3*], Antonio Rossi [1,2], Lamar Elfaki [4], Mavra Ahmed [1,5], Victoria Chen [1,2], Shuting Yang [1,2], Andreea Zurbau [1,2,6], Alison M Duncan [7], Cyril WC Kendall [1,2,8], John L Sievenpiper [1,2,9-11], and Laura Chiavaroli [1,2,11*]

1 Department of Nutritional Sciences, Temerty Faculty of Medicine, University of Toronto, Toronto, Ontario, Canada
2 Toronto 3D Knowledge Synthesis and Clinical Trials Unit and Clinical Nutrition and Risk Factor Modification Centre, St. Michael's Hospital, Toronto, Ontario, Canada
3 Center for Regulatory Research and Innovation, Protein Industries Canada, Regina, Saskatchewan, Canada
4 Faculty of Arts and Science, University of Toronto, Toronto, Ontario, Canada
5 Joannah & Brian Lawson Centre for Child Nutrition, Temerty Faculty of Medicine, University of Toronto, Toronto, Ontario, Canada
6 INQUIS Clinical Research Ltd. (formerly GI Labs), Toronto, Ontario, Canada
7 Department of Human Health and Nutritional Sciences, University of Guelph, Guelph, ON, Canada
8 College of Pharmacy and Nutrition, University of Saskatchewan, Saskatoon, Saskatchewan, Canada
9 Division of Endocrinology and Metabolism, Department of Medicine, St. Michael's Hospital, Toronto, Ontario, Canada
10 Department of Medicine, Temerty Faculty of Medicine, University of Toronto, Toronto, Ontario, Canada
11 Li Ka Shing Knowledge Institute, St. Michael's Hospital, Toronto, Ontario, Canada

Table S1. Identification of Health Canada's BNS Food Group Codes that contain mainly plant derived foods with protein.[1]

Plant BNS Food Group Codes with Foods that Contain Protein Primarily Derived from Plants			
**BNS Food Group Codes**	**BNS Food Group Description**	**BNS Food Group Codes**	**BNS Food Group Description**
01 A-C	Pasta, rice, cereal grains and flour	38 A-B	Potatoes, fried
02 A	White breads	39 A	Potato (excluding fried)
03 A-B	Wholemeal breads	40 A-L	Fruits, raw, cooked, frozen and canned
04 A-F	Other breads	41 A-D	Sugars, syrups, and preserves
05 A	Whole grain and high fibre breakfast cereals	42A-B	Savory snacks
06 A	Breakfast cereal (other)	43 A-B	Confectionary -candy/gum, popsicle, sherbert
07 A-B	Cookies, biscuits and granola bars	44 A	Confectionery, chocolate bars
08 A-C	Cakes, pies, danishes and other pastries, commercial	45 A	Fruit juices
10 J	Milks – plant-based beverage (soy, almond, coconut)	50 D-E	Miscellaneous foods (sauces, salad dressings, seasoning)
18 A-B	Margarines (tub)	51 B	Coffee
33 A-C	Nuts, seeds and peanut butter	53 A-B	Other recipe ingredients
36 A-P	Vegetables (excluding potatoes)	54 C	Meal replacements
37A-B	Legumes		

Abbreviations: BNS, Bureau of Nutritional Sciences (Health Canada).
Table S1 was adopted from Marinangeli et al. [1]
[1]BNS Food Codes in Table S1 have not been changed from the list published by Health Canada, although modifications (e.g. expansion of subcategories within a code) were made as per Table S2.

Table S2. Removal and/or reclassification of food items in the Canadian Nutrient File for determining the protein quality of BNS food groups and eligibility for protein content claim under regulatory frameworks for Canada, the US, ANZ and the EU.

Category of Assumption	Description of the Change	BNS Food Groups Affected	Explanation	Reference (where applicable)
1. Food item additions to the 2018 CNF	94 food items from the 2015 Canadian Nutrition File were added to the 2018 Canadian Nutrient File used in this study.		Missing food items from the 2015 Canadian Nutrient File were added to the 2018 Canadian Nutrient File to ensure nutrient intakes from the Canadian Community Health Survey - 2015 could be considered in the analysis.	Marinangeli et al. [1]: Supplementary Table S1
2. Removal of food items from the CNF dataset	1921 foods with incomplete protein and amino acid data were removed from the dataset based on previous analysis.<br><br>Additional foods were removed based assumptions in Supplementary Table S3 for this study.		Data would falsely reduce the protein and/or IAA levels of a BNS food group for protein quality and eligibility for a protein content claim.	Marinangeli et al. [1]: Supplementary Table S3
3. Food Item Recategorization	Sweet potatoes and yams with complete protein and IAA data were re-classified from “Other Veg (cucumber, immature beans, Brussel sprouts, beets, turnips)” (BNS Group: 36P) to the “Potato” group (BNS Group: 39A).	36P Other vegetables (cucumber, immature beans, brussel sprouts, beets, turnips)<br>39A Potato	More accurate determination of how these foods were consumed.	Marinangeli et al. [1]: Supplementary Table S4
4. New BNS food group creation and food item recategorization	Foods in the “plant-based beverage” group (BNS 10J) were further segregated and re-classified as soy-based beverages (BNS 10J) and almond-based beverages (BNS 10L).	10J Soy-based beverages<br>10L Almond-based beverages (new group)	Food products are manufactured using ingredients with significantly different protein and IAA levels.	
5. New BNS food group creation and food item recategorization	Foods in the “cookies, commercial” group (BNS food group 07A, all types of cookies, granola bars, breakfast and energy bars) were segregated and reclassified as cookies, commercial (7A) and granola bars (07C).	07A Cookies, commercial<br>07C Granola bars (new group)	Food products are manufactured using ingredients with significantly different protein and IAA levels.	

6. New BNS food group creation and food item recategorization	Based on previous analysis as well as consideration in the present study, BNS Food Group 37 (legumes) was further segregated into newly created BNS subgroups based on differences in protein, protein quality, and IAA levels.	Creation of BNS Food Groups:<br>37A: Dry beans<br>37B: legume (soy-based)<br>37C: sprouted legumes (non-soy)<br>37D sprouted legumes (soy)<br>37E Meat Alternatives (unidentified protein source)<br>37F Chickpeas<br>37G Lentils<br>37H Peas		
7. Food Item Recategorization	CNF Code 6918, "ready to eat, Force Active, President's Choice"	Food was moved from BNS Food Group 05A (Whole Grain/Oats/High Fibre Breakfast Cereals) to 54C (Meal Replacement)	This food product is considered to be a meal replacement.	
8. Food Item Recategorization	CNF Code 3320 "Soybean, fermented products, natto"	Food item was moved from BNS Food Group 50F "Seasonings" to BNS Food Group 37B Legume (soy-based)	Food format is better aligned with BNS Group 37B (legume (soy-based)) based on how the food is used in dietary patterns.	
9. Food Item Recategorization	CNF Code 2081 "Beans, yardlong, (asparagus bean or cowpea), boiled, drained" were moved from 37A to 36A.<br><br>CNF Code 2080 "Beans, yardlong, (asparagus bean or cowpea), raw" were moved from 37A to 36A.<br><br>CNF Code 6559 "Yardlong beans (asparagus bean or cowpea), boiled, drained, with salt" were moved from 37A to 36A.	Food items were moved from 37A (dry beans) to 36A beans	Food format is better aligned with BNS Group 36A (Beans) as this bean is not dried on the vine as per the definition of a pulse crop and is more aligned as a vegetable in dietary patterns.	

Abbreviations: BNS, Bureau of Nutritional Sciences; CNF, Canadian Nutrient File; IAA, indispensable amino acids

Table S3. Specific food items that were originally in BNS Food Group 37A Legume (Non-Soy) and recategorized to the newly created BNS Food Group 37A Beans.

Food Item Name	BNS Food Code
Beans, adzuki	
Beans, adzuki, boiled	3244
Beans, adzuki, boiled, salted	3340
Beans, adzuki, canned, sweetened	3245
Beans, adzuki, yokan (bean jelly)	3246
Beans, baked, canned, plain or vegetarian	3248
Beans, black turtle	
Beans, black turtle, mature seeds, boiled	3253
Beans, black turtle, mature seeds, boiled, salted	3342
Beans, black turtle, mature seeds, canned, solids and liquid	3254
Beans, black	
Beans, black, mature seeds, boiled	3377
Beans, black, mature seeds, boiled, salted	3341
Beans, black, mature seeds, canned, solids and liquid, reduced sodium	7235
Beans, cranberry (roman)	
Beans, cranberry (roman), boiled	3256
Beans, cranberry (roman), boiled, salted	3343
Beans, cranberry canned, with solids and liquid	3378
Beans, great northern	
Beans, great northern, boiled	3260
Beans, great northern, boiled, salted	3345
Beans, great northern, canned, solids and liquid, no salt added	3261
Beans, great northern, canned, solids and liquid, reduced sodium	7236
Beans, hyacinth	
Beans, hyacinth, boiled	3287
Beans, hyacinth, boiled, salted	3355
Beans, kidney, all types	
Beans, kidney, all types, boiled	6366
Beans, kidney, all types, boiled, with salt	6365
Beans, kidney, all types, canned, solids and liquid	3379
Beans, kidney, dark red	
Beans, kidney, dark red, boiled	3382
Beans, kidney, dark red, boiled, salted	3407
Beans, kidney, dark red, canned, solids and liquid	3265
Beans, kidney, light red	
Beans, kidney, light red, boiled	3264
Beans, kidney, light red, boiled, salted	3347
Beans, kidney, red	
Beans, kidney, red, canned, drained and rinsed	7085
Beans, kidney, red, canned, drained solids	7081
Beans, kidney, red, canned, solids and liquid, reduced sodium	7240
Beans, legumes, mung (green gram)	
Beans, legumes, mung (green gram), boiled	3298
Beans, legumes, mung (green gram), boiled, salted	3360
Beans, lima (fordhook)	
Beans, lima (fordhook), frozen, boiled, drained	2007
Beans, lima (fordhook), frozen, boiled, drained, with salt	6455
Beans, lima, baby	
Beans, lima, baby, frozen, boiled, drained	2369
Beans, lima, baby, frozen, boiled, drained, with salt	6454
Beans, lima, dry, baby, boiled	3292
Beans, lima, dry, baby, boiled, salted	3357
Beans, lima	
Beans, lima, boiled, drained	2004
Beans, lima, boiled, drained, with salt	6453
Beans, lima, canned, solids and liquid	2005
Beans, lima, large	
Beans, lima, dry, large, boiled	3289
Beans, lima, dry, large, boiled, salted	3356

Beans, lima, dry, large, canned, solids and liquid	3290
Beans, mungo	
Beans, mungo, boiled	3301
Beans, mungo, boiled, salted	3361
Beans, navy	
Beans, navy, boiled	3384
Beans, navy, boiled, salted	3408
Beans, navy, canned, solids and liquid	3266
Beans, pink	
Beans, pink, boiled	3268
Beans, pink, boiled, salted	3348
Beans, pinto	
Beans, pinto, boiled	3270
Beans, pinto, boiled, salted	3349
Beans, pinto, canned, drained and rinsed	7086
Beans, pinto, canned, drained solids	7083
Beans, pinto, canned, solids and liquid	3271
Beans, pinto, canned, solids and liquid, reduced sodium	7241
Beans, small white	
Beans, small white, boiled	3273
Beans, small white, boiled, salted	3350
Beans, white	
Beans, white, boiled	3386
Beans, white, boiled, salted	3409
Beans, white, canned, solids and liquid, no salt added	3276
Beans, winged (goa beans)	
Beans, winged (goa beans), dry, boiled	3337
Beans, winged (goa beans), dry, boiled, with salt	3373
Beans, yellow	
Beans, yellow, boiled	3275
Beans, yellow, boiled, salted	3351
Broadbeans (fava beans)	
Broadbeans (fava beans), canned, solids and liquid	3277
Broadbeans (fava beans), dry, boiled	3388
Broadbeans (fava beans), dry, boiled, with salt	3352
Broadbeans (fava beans), fresh, boiled, drained	2024
Broadbeans (fava beans), fresh, boiled, drained, with salt	6385
Beans, french	
Beans, french, mature seeds, boiled	3258
Beans, french, mature seeds, boiled, salted	3344
Beans, yardlong, mature seeds	
Beans, yardlong, mature seeds, boiled	3335
Beans, yardlong, mature seeds, boiled, salted	3372
Black-eyed peas (cowpeas, crowder, southern), common, boiled, salted	3354
Black-eyed peas (cowpeas, crowder, southern), common, mature seeds	
Black-eyed peas (cowpeas, crowder, southern)), common, mature seeds, boiled	3283
Black-eyed peas (cowpeas, crowder, southern), common, mature seeds, canned, plain, solids and liquid	3284
Black-eyed peas (cowpeas), catjang	
Black-eyed peas (cowpeas), catjang, boiled	3281
Black-eyed peas (cowpeas), catjang, boiled, salted	3353
Cowpeas, boiled, drained, with salt	2333
Lupins	
Lupins, boiled	3294
Lupins, boiled, salted	3358
Refried beans	
Refried beans	7449
Refried beans, canned	3314
Refried beans, canned, reduced sodium	7243

Table S4. Specific food items that were originally in BNS Food Group 37A Legume (Non-Soy) and recategorized to the newly created BNS Food Group 37F Chickpeas

Food Item Name	BNS Food Code
Chickpeas (garbanzo beans, bengal gram), boiled	3390
Chickpeas (garbanzo beans, bengal gram), boiled, salted	3410
Chickpeas (garbanzo beans, bengal gram), canned, drained solids	7060
Chickpeas (garbanzo beans, bengal gram), canned, drained, rinsed	7061
Chickpeas (garbanzo beans, bengal gram), canned, solids and liquid	3279
Chickpeas (garbanzo beans, bengal gram), canned, solids and liquid, reduced sodium	7242

Table S5. Specific food items that were originally in the BNS Food Group 37A Legume (Non-Soy) and recategorized to the newly created BNS Food Group 37G Lentils.

Food Item Name	BNS Food Code
Lentils, boiled	3393
Lentils, boiled, salted	3411
Lentils, pink, boiled	5918

Table S6. Specific food items that were originally in the BNS Food Group 37A Legume (Non-Soy) and recategorized to the newly created BNS Food Group 37H Dried Peas.

Food Item Name	BNS Food Code
Peas, pigeon (red gram)	
Peas, pigeon (red gram), boiled	3313
Peas, pigeon (red gram), boiled, salted	3367
Peas, split	
Peas, split, boiled	3395
Peas, split, boiled, salted	3412

Table S7. Summary and rationale for excluding or including plant BNS food groups and/or food items from the 2018 Canadian Nutrient File for determining the protein quality of plant BNS food groups and eligibility for protein content claim under regulatory frameworks for Canada, the US, ANZ and the EU.

Assumption Category	Reason for Exclusion of food items	BNS Food Groups Affected		Rationale for Exclusion	Reference (where applicable)
1. Food Item Exclusion	Food items in plant BNS food groups that contained animal proteins were excluded from protein quality and protein content claim assessments.	01A Pasta<br>02A White Breads<br>04A Rolls/Bagels/Pita/Croutons/ Dumplings/Matzo/ Tortilla<br>04B Crackers/Crispbread<br>4C Muffins/English Muffins<br>4D Pancakes/Waffle<br>4E Croissants/Piecrusts/Phyllo Dough<br>4F Dry Mixes - Cakes/Muffins/ Pancakes<br>7B Biscuits	8A Pies<br>8B Cakes<br>8C Danishes/Doughnuts/Other Pastries<br>36O Juices, Tomato &Vegetable<br>39A Potato<br>42B Salty/High-Fat Snacks<br>44A Chocolate Bar<br>50D Sauces<br>51B Coffee	Prevent inflation of protein quality for BNS food group. Permitted a more conservative estimate of protein quality	
2. Food Item Exclusion	Food items in plant-based BNS food groups that were combination dishes were excluded from protein quality and protein content claims assessments.<br><br>A "combination dish" has been defined in Canadian Food and Drug Regulations, as foods that do **not require** the addition of ingredients, other than water, for its preparation and that contains f**ood from at least two of the following categories**: dairy products, meat products, fruits and vegetables or breads. Some exceptions apply.	01A Pasta<br>01B Rice<br>04A Rolls/Bagels/Pita/Croutons/ Dumplings/Matzo/Tortilla<br>36B Broccoli<br>36E Carrots<br>36G Corn<br>36I Mushrooms	36J Onion/Green Onions/ Leeks/Garlic<br>36M Squashes<br>36N Tomatoes<br>36P Other Vegetables<br>37A Dry Beans<br>37F Chickpeas<br>37B Legume - Soy Based<br>39A Potato	Prevent inflation of protein quality for BNS food group. Permitted a more conservative estimate of protein quality	Government of Canada. [2]
3. Food Item Exclusion	If similar food items in a BNS food group were present, but food items were in an unsuitable form of consumption (i.e. dry beans), they were excluded form protein quality and protein content claims assessments.	33A Nuts<br>33B Seeds<br>37A Dry beans<br>37F Chickpeas<br>37G lentils<br>37H Dry Peas<br>39A Potato		Does not reflect how foods are consumed. In the case of foods that require hydration (i.e., dry beans) the protein level would be inflated.	

4. BNS Food Group Exclusion	A BNS food group was considered to be a negligible source of protein if the median protein amount per 100 g serving was 0. These BNS food groups were excluded from protein quality and protein content claims assessments.	21A Vegetable Oils<br>21C Shortening<br>41A Sugars - White/Brown<br>41C Other Sugars<br>41D Sugar Substitutes<br>43B Popsicle/Sherbet<br>46A Soft Drinks - Regular<br>46B Soft Drinks - Diet<br>46 C Fruit Drinks	46F Vitamin Water<br>46G Sports Drink<br>47A Spirits<br>47B Liqueurs<br>49A Beer<br>50F Seasonings<br>51A Tea<br>51C Water	Plant BNS groups that did not contain any protein do not provide protein to dietary patterns.	
5. Food Item Exclusion	Protein isolates/concentrates food items were excluded from protein quality and protein content claims assessments for BNS foods groups.	37B Legume - Soy Based,<br>54C Meal Replacements		Protein isolates and concentrates are considered to be Natural Health Products.	
6. Food Item Exclusion to remove duplicates	Food items in BNS food groups that were identified to be prepared differently than what is outlined in the Table of Reference Amounts for Food was excluded from protein quality assessments and/or protein content claim assessments for the corresponding BNS food group.<br><br>If the same food (a duplicate) was present with a method of preparation outlined in Health Canada's Table of Reference Amounts for Food" it remained in the analysis. This removed food duplicates<br><br>However, for some food some items, protein and amino acid data was not available for prepared versions of the food. In these cases, the unprepared food item that had protein and amino acid data remained in the protein quality and protein content claims assessments. For example, BNS 1C, dry grains data was included if the cooked version was not available.	01A Pasta<br>1B Rice<br>1C Cereal/Grains/Flour<br>5A Whole Grain/Oats/High Fibre Breakfast Cereals<br>6A Breakfast Cereal - Other<br>51B Coffee		Prevent invalid weighting of protein content in the BNS food group. Permitted a more conservative estimate of protein quality for the BNS food group	Government of Canada. [3]
7. Food Item Inclusion	If a food item in a BNS food group was prepared in an unsuitable form for consumption (i.e., uncooked pasta), it was included in the protein quality and protein content claim assessment if the same type of food item was not present in a prepared format.	01A Pasta<br>01B Rice<br>39A Potato		Ensure the contribution of these food items were captured in protein quality and eligibility for protein content claim assessments.	

8. Food Item Inclusion	If BNS food groups for Vegetables (BNS Code 36) or fruit (BNS Code 40) only contained one specific type of food, all methods of preparation were included to assess the protein quality and eligibility for a protein content claim of the BNS food group.	36A Beans<br>36B Broccoli<br>36D Cauliflower<br>36E Carrots<br>36F Celery<br>36G Corn<br>36I Mushrooms<br>36L Peppers - Red/Green<br>36N Tomatoes	38A Potato Chips<br>40B Apple<br>40C Banana<br>40D Cherries<br>40E Grapes/Raisins<br>40G Peaches/Nectarines<br>40H Pears<br>40I Pineapple<br>40J Plums/Prunes	This reflects utilization of foods by consumers	
9. Food Item Inclusion	Some foods in plant BNS food groups contained animal-derived fat (e.g. Butter). If the protein was derived from plant-based sources, food items with animal-based fats, such as butter, was included in the protein quality assessment for the BNS food group.	Applied to all BNS Food Groups where applicable.		Animal-derived fat would not affect the contribution of plant ingredients to the protein and amino acid content of foods.	
10. Aggregated methods of preparation	Some foods are consumed using multiple methods of preparation. If a food item was present in the BNS food group, with multiple methods of preparation (e.g. roasted, baked, fried), the average protein and indispensable amino acid content across foods was used in protein content claim assessments.	33A Nuts<br>33B Seeds<br>33C Peanut Butter/ Other Nut Spreads<br>36C Cabbage/Kale<br>36H Lettuce/Leafy Greens<br>36J Onion/Green Onions/Leeks/Garlic<br>36M Squashes<br>36P Other Vegetables	37A Dry beans<br>37F Chickpeas 37 G lentils<br>37H Dry Peas<br>37B Legume - Soy Based<br>37C Sprouted Legumes - Non-Soy<br>39A Potato	Prevented one type of food from being weighted too heavily and skewing the median protein levels and amino acid levels for protein content claim assessments.	

Abbreviations: BNS, Bureau of Nutritional Sciences; CNF, Canadian Nutrient File

Table S8. Assumptions and data used to calculate the protein quality and eligibility for protein content claims for plant BNS Food Groups under regulatory frameworks for Canada, the US, Australia and New Zealand and the EU.

Assumption Category	Assumption	BNS Food Groups Affected	Explanation	Reference (where applicable)
PDCAAS Assessment (US and Canada (when PER was not available))	When ≤ 2 true N coefficients were available for foods included in a BNS food group, a conservative coefficient of 0.8 was used for PDCAAS calculations.	01B Rice<br>06A Breakfast Cereal - Other<br>33B Seeds<br>33C Peanut Butter/ Other Nut Spreads<br>36G Corn<br>36K Peas/Snow Peas<br>37B Legume - Soy	Eliminate a potential overestimation for digestibility	
PDCAAS Assessment (US and Canada (when PER was not available))	When > 2 true N coefficients were available for foods included in a BNS food group, the mean true N digestibility coefficient was used.	1C Cereal<br>33A Nuts<br>37A Dry Beans<br>37F Chickpeas<br>37G Lentils<br>37H Dried peas	Adequately accurate assumption of true N digestibility	See Table S10
PDCAAS Assessment (US and Canada (when PER was not available))	If a true N coefficient for a food in BNS food group were not available, a conservative coefficient of 0.8 was used for PDCAAS calculations.	All plant BNS food groups without a true N digestibility coefficient	Eliminate a potential overestimation for digestibility	Marinangeli et al. [1]
Protein Quality Assessments (Canada)	For protein quality assessments under the Canadian framework, the protein from Reasonable Daily Intake (Schedule K - Food and Drug Regulations) for foods within the BNS food group was used.<br><br>However, if a Reasonable Daily Intake for a food was not available, the Reference Amount was used.	All plant BNS food groups	Health Canada and the Canadian Food Inspection Agency have specified that if a Reasonable Daily intake is not published in Schedule K of Canada's Food and Drug Regulations, the Reference Amount can be used for protein quality calculations.	Government of Canada. [4]<br><br>Canadian Food Inspection Agency. [5]<br><br>Canadian Food Inspection Agency. [6]

Reasonable Daily Intake for Breakfast Cereals (Canada)	For protein quality assessments under the Canadian framework, the appropriate Reasonable Daily Intake level as per Schedule K of Canada's Food and Drug Regulation.<br><br>For puffed-type breakfast cereals, 14g was used.<br><br>For all other unprepared breakfast cereals, 28g was used.<br><br>For prepared breakfast cereals (i.e. oatmeal), the reference amount was used.	5A Whole Grain/Oats/High Fibre Breakfast Cereals<br>6A Breakfast Cereal - Other<br>54C Meal Replacement	Alignment with protein content claim regulations at the time of the analysis.	Government of Canada. [7]<br>Government of Canada. [3]
Reference Amount for ready-to-eat breakfast Cereals (Canada, US, EU, and ANZ)	To determine eligibility for a protein content claim for breakfast cereals under Canada (prepared breakfast cereals only), US, ANZ, and EU frameworks, the reference amount used corresponded to values in Health Canada's Table of Reference Amounts for Food.<br><br>Hot breakfast cereals, such as oatmeal, or cream of wheat: 250 ml prepared (converted to grams)<br><br>Ready-to-eat breakfast cereals, puffed and uncoated (weighing less than 20 g per 250 mL): 15g<br><br>Ready-to-eat breakfast cereals, puffed and coated, flaked, extruded, without fruit or nuts (weighing 20 g to 42 g per 250 mL), very high fibre cereals (with 28 g or more fibre per 100 g): 30g	5A Whole Grain/Oats/High Fibre Breakfast Cereals<br>6A Breakfast Cereal - Other<br>54C Meal Replacement	Alignment with protein content claim regulations at the time of the analysis.	Government of Canada. [3]
Reasonable Daily Intake (Canada – where applicable) and Reference Amounts for Dry Mixes, Potato, and Coffee (Canada, US, EU, and ANZ)	For protein quality and protein content claim assessments, the Reasonable Daily Intake and Reference amount for dry mixes, instant-type potatoes, and coffee were derived from cooked forms of the foods.	04F Dry Mixes<br>39A Potato<br>50E Coffee	The amount of dry mix powder corresponds to the weight of final product. It is unreasonable to reconstitute all foods/products to configure the exact dry-to-prepared conversions, and the dry mix amount already closely corresponds to the reference amount (based on the serving sizes of the products in the marketplace; some variations are due to the use of household measures).	

Abbreviations: ANZ, Australia and New Zealand; BNS, Bureau of Nutritional Sciences; EU, European union; PDCAAS, Protein digestibility corrected amino acid score; PER, protein efficiency ratio; US, United States

Table S9. Summary of Protein Efficiency Ratios used to calculate mean PER for plant BNS food groups under the Canadian regulatory framework for protein content claims.[1]

BNS Food Group	Food with an Identified PER*	PER	MEAN PER
BNS 1B: Rice	Rice	1.5	1.5
BNS 1C: Cereal/Grains/Flour	Oats, rolled	1.8	1.27
	Barley	1.7	
	Bulgar wheat	1.4	
	Rye	1.3	
	Pea flour	1.2	
	White flour	0.7	
	Wheat, whole	0.8	
BNS 2A; White Breads	Bread, white	1.0	1.0
BNS 10J: Plant-Based Beverages (Soy)	Soybean, heated	2.3	2.3
BNS 10L: Plant-Based Beverages (Non-Soy)	Almonds	0.4	0.4
BNS 33A: Nuts	Peanuts	1.7	1.05
	Almonds	0.4	
BNS 33B: Seeds	Sunflower Seed	1.2	1.2
BNS 33C: Peanut Butter/Other Nut Spreads	Peanuts	1.7	1.1
	Almonds	0.4	
	Sunflower Seed	1.2	
BNS 36G: Corn	Corn, whole	1.4	1.4
BNS 36K: Peas/Snow Peas	Pea, split yellow	1.4	1.4
BNS 37A: Legume (Non-Soy)	Chickpea, cooked	2.32	1.46
	Bean, pinto	1.6	
	Bean, black	1.6	
	Bean, kidney	1.6	
	Bean, navy (dry)	1.5	
	Lentil, whole green	1.3	
	Lentil, cooked (all others)	0.3	
BNS 37B: Legume (Soy)	Soybean, heated	2.3	2.3

BNS 37D: Sprouted Legumes (Soy)	Soybean, heated	2.3	2.3
BNS 37A: Beans	Bean, pinto<br>Bean, black<br>Bean, kidney<br>Bean, navy (dry)	1.6<br>1.6<br>1.6<br>1.5	1.58
BNS 37F: Chickpeas	Chickpea, cooked	2.32	2.32
BNS 37G: Lentils	Lentil, whole green<br>Lentil, cooked (all others)	1.3<br>0.3	0.8
BNS 37H: Dried Peas	Pea, split yellow	1.4	1.4

Abbreviations: BNS, Bureau of Nutritional Science; PER, Protein Efficiency Ratio
[1]All PER values are published by the Canadian Food Inspection Agency [6]

Table S10. Summary of true nitrogen digestibility coefficients derived from the literature and applied to plant BNS food groups for PDCAAS calculations.

Relevant BNS Food Group	Food with a True Nitrogen Digestibility Coefficient	True Nitrogen Digestibility Coefficient	Reference	Mean TNDC
BNS 1C: Cereal	Rice, cereal	75	FAO [8]	87.45
	Corn, cereal	70	FAO [8]	
	Wheat, cereal	77	FAO [8]	
	Oats, cereal	72	FAO [8]	
	Sunflower seed, flour	90	FAO [8]	
	Wheat, whole	86	FAO [8]	
	Wheat, refined	96	FAO [8]	
	Wheat flour, white	96	FAO [8]	
	Wheat gluten	99	FAO [8]	
	Oatmeal	86	FAO [8]	
	Millet	79	FAO [8]	
	Soy flour	86	FAO [8]	
	Pea flour	88	FAO [9]	
	Wheat	93	FAO [9]	
	Peanut meal	91	FAO [9]	
	Sunflower meal	90	FAO [9]	
	Rolled Oats	94	FAO [9]	
	Red lentil flour, extruded	92.38	Nosworthy et al. [10]	
	Red lentil flour, cooked	90.95	Nosworthy et al. [10]	
	Red lentils flour, baked	88.8	Nosworthy et al. [10]	
	Green lentil flour, extruded	96.2	Nosworthy et al. [10]	
	Green lentil flour cooked	86.42	Nosworthy et al. [10]	
	Green lentil flour, baked	83.05	Nosworthy et al. [10]	
	Cooked rolled oats	95.2	Rutherfurd et al. [11]	
	Wheat bran	85.3	Rutherfurd et al. [11]	
BNS 33A: Nuts	Peanuts	94	FAO [8]	92.56
	Peanut butter	95	FAO [8]	
	Peanut	96	FAO [9]	
	Almond, Independence	88.9	House et al. [12]	
	Almond, Monterey	89.9	House et al. [12]	
	Almond, Nonpareil	85.7	House et al. [12]	
	Roasted peanuts	98.4	Rutherfurd et al. [11]	
BNS 37A: Beans	Faba bean (autoclaved)	86	FAO [9]	79.08
	Pinto bean (canned)	79	FAO [9]	

	Red Kidney Beans	78.6	Nosworthy et al. [13]	
	Navy Beans	79.96	Nosworthy et al. [13]	
	Black beans	69.99	Nosworthy et al. [13]	
	Pinto beans	76.23	Nosworthy et al. [13]	
	Faba bean, extruded	87.6	Nosworthy et al. [14]	
	Faba bean, cooked	88.49	Nosworthy et al. [14]	
	Faba bean, baked	88.63	Nosworthy et al. [14]	
	Black bean, extruded	82.01	Nosworthy et al. [14]	
	Black bean, cooked	81.66	Nosworthy et al. [14]	
	black bean baked	63.55	Nosworthy et al. [14]	
	Navy bean, extruded	87.41	Nosworthy et al. [14]	
	Navy bean, cooked	86.02	Nosworthy et al. [14]	
	Navy bean, baked	69.08	Nosworthy et al. [14]	
	Pinto bean, extruded	82.53	Nosworthy et al. [14]	
	Pinto bean, cooked	82.07	Nosworthy et al. [14]	
	Pinto bean, baked	57.58	Nosworthy et al. [14]	
	Red Kidney bean, extruded	83.21	Nosworthy et al. [14]	
	Red Kidney bean, cooked	80.67	Nosworthy et al. [14]	
	Red Kidney bean, Baked	69.12	Nosworthy et al. [14]	
	Cooked Kidney Beans	80.4	Rutherfurd et al. [11]	
BNS 37F: Chickpeas*	Chickpeas	85.02	Nosworthy et al. [13]	85.02
BNS 37G: Lentils	Lentil (autoclaved)	85	FAO [9]	87.83
	Whole bean lentils	87.89	Nosworthy et al. [13]	
	Split red lentils	90.6	Nosworthy et al. [13]	
BNS 37H: Dried Peas	Split yellow peas	87.94	Nosworthy et al. [13]	88.38
	Split green peas	85.15	Nosworthy et al. [13]	
	Yellow pea, extruded	91.35	Nosworthy et al. [15]	
	Yellow pea, cooked	89	Nosworthy et al. [15]	
	Yellow pea, baked	86.77	Nosworthy et al. [15]	
	Green pea, extruded	90.73	Nosworthy et al. [15]	
	Green pea, cooked	87.58	Nosworthy et al. [15]	
	Green pea, baked	88.51	Nosworthy et al. [15]	

Abbreviations: BNS, Bureau of Nutritional Sciences
*For protein quality analysis a 0.8 true nitrogen digestibility coefficient was used given that digestibility data for ≤ 2 chickpea foods were identified.

Table S11. Indispensable amino acid reference patterns used to determine PDCAAS of plant BNS food groups for eligibility of PCC under the US framework and DIAAS for diets.

Age Group	Indispensable Amino Acid Reference Scoring Requirement (mg/g protein)								
	**Protein Digestibility Corrected Amino Acid Calculations[1] (Section 2.2.2)**								
	**HIS**	**ILE**	**LEU**	**LYS**	**SAA (MET + CYS)**	**AAA (PHE + TYR)**	**THR**	**TRP**	**VAL**
**Pre-school child (2-5 years)**	19	28	66	58	25	63	34	11	35
	**Digestible Indispensable Amino Acid Score Calculations[2] (Section 2.4)**								
	**HIS**	**ILE**	**LEU**	**LYS**	**SAA (MET + CYS)**	**AAA (PHE + TYR)**	**THR**	**TRP**	**VAL**
**Older child, adolescents, and adults**	16	30	61	48	23	41	25	6.6	40

Abbreviations: AAA, aromatic amino acids; CYS, cysteine/cystine; HIS, histidine; ILE, isoleucine; LEU, leucine; LYS, lysine, MET, methionine; PHE, phenylalanine; SAA, sulfur amino acids; THR, threonine; TRP, tryptophan; TYR, tyrosine; VAL, valine

[1] FAO [16]

[2] From FAO [17]

Table S12. Summary of BNS food groups identified as containing food items that provided plant protein, animal protein, mixture of both or negligible amounts of protein.

**BNS Food Groups that Contained Plant Protein or Mixture of Plant and Animal Protein n = 77**		**BNS Food Groups that Contained Animal Protein n=59**		**Negligible protein or No Information on Protein and/or Amino Acid Content n = 27**
**Plant Protein (n = 59)**				**Negligible Protein (n = 17)**
01B Rice	37C Sprouted Legumes (Non-Soy)	09A Ice Cream	24B Lamb, Lean + Fat/Ground	21A Vegetable Oils
01C Cereal/Grains/Flour	37D Sprouted Legumes (Soy)	09B Ice Milk	25A Pork, Fresh - Lean Only	21B Animal Fats
03A Whole Wheat Breads	37E Meat Alternatives (Unidentified Protein Source)	09C Frozen Yoghurt	25B Pork, Fresh - Lean + Fat/Ground	21C Shortening
03B Other Whole Grains Breads		10A Milk, Whole	25C Bacon	41A Sugars, White/Brown
05A Whole Grain/Oats/High Fibre Breakfast Cereals	37F Chickpeas	10B Milk, 2%	25D Ham, Cured - Lean Only	41C Other Sugars (Syrups/Molasses/Honey)
	37G Lentils	10C Milk, 1%	25E Ham, Cured - Lean + Fat	41D Sugar Substitutes
06A Breakfast Cereal (Other)	37H Dried Peas	10D Milk, Skim	27A Chicken, Meat Only	43B Ice Pop/Sherbet
07A Cookies, Commercial	38A Potato Chips	10E Milk, Evaporated Whole	27B Chicken, Meat + Skin	46A Soft Drinks, Regular
07C Granola Bar	38B Fried/Roasted Potatoes	10G Milk, Evaporated Skim	27C Turkey, Meat Only	46B Soft Drinks, Diet
10J Plant-Based Beverage (Soy)	40A Citrus Fruit (Oranges/Lemons/Grapefruits)	10H Milk, Condensed	27D Turkey, Meat + Skin/Ground	46C Fruit Drinks
10L Plant-Based Beverage (Non-Soy)	40B Apple	10I Milk, Other (Whey/Buttermilk)	27E Other Birds (Duck/Pheasant/Pigeon)	46F Vitamin Water
18A Regular Margarine	40C Banana	10K Milk : Goat/Sheep	27F Birds, Skin Only	46G Sports Drink
33A Nuts	40D Cherries	13A Whipping Cream	28A Liver	47A Spirits
33B Seeds	40E Grapes/Raisins	13B Table Cream	28B Liver Pâté	49A Beer
33C Peanut Butter/Other Nut Spreads	40F Melons (Cantaloupe/Honeydew/Watermelon)	13C Half & Half Cream	29A Offal	50F Seasonings (Salt/Vinegar)
36A Beans		13D Sour Cream	30A Sausage	51A Tea (Incl Iced Tea)
36B Broccoli	40G Peaches/Nectarines	14A Cottage Cheese	31A Game Meat	51C Water (Well/Mineral)
36C Cabbage/Kale	40H Pears	14B Cheese, Less than 10% B.F.	32A Luncheon Meat	
36D Cauliflower	40I Pineapple	14C Cheese, 10% B.F. to 25% B.F.	34A Fish, Less than 6% Total Fat	**No Information (n = 10)**
36E Carrots	40J Plums/Prunes	14D Cheese, More than 25% B.F.	34B Fish, $\geq$ 6% Total Fat	10F Milk, Evaporated 2%
36F Celery	40K Strawberries	15A Yoghurts, Less than 2% B.F.	35A Shellfish	18B Calorie Reduced Margarine
36G Corn	40L Other Fruits (Blueberries/Date/Kiwi/Fruit Salad)	15B Yoghurts, More than 2.1% B.F.	43C Gelatin/Dessert Toppings/Pudding Mixes, Commercial	20A Block Margarine
36H Lettuce/Leafy Greens (Spinach/Mustard Greens)		16A Egg		46E Energy Drink
	41B Jams/Jellies/Marmalade	16B Egg Substitutes	46D Other Beverages (Malted Milk/Chocolate beverage)	48A Wine
36I Mushrooms	42A Plain Popcorn/Pretzels	17A Butter		49B Coolers
36J Onion/Green Onions/Leeks/Garlic	43A Candy/Gum	22A Beef, Lean Only	47B Liqueurs	50C Gravies
36K Peas/Snow Peas	45A Fruit Juice	22B Beef, Lean + Fat	50A Soups with Vegetables	52B Infant Formula

36L Peppers, Red/Green	50E Salad Dressings (With or Without Oil)	22C Beef, Ground	50B Soups without Vegetables	54A Energy Bar
36M Squashes	53A Spices	23A Veal, Lean Only	52A Baby food Product	54B Protein Bar and Shake
36N Tomatoes	53B Others (Baking Soda/Baking Powder/Yeast)	23B Veal, Lean + Fat/Ground	99A Mexican Recipes	
36P Other Veg. (Cucumber/Beet/Turnip)	54C Meal Replacements	24A Lamb, Lean Only		
37A Beans				
37B Legume (Soy-Based)				
**Mixture of Plant and Animal Proteins (n=18)**				
01A Pasta	08B Cakes, Commercial (Frozen Cake)			
02A White Breads	08C Danishes/Doughnuts/Other Pastries, Commercial			
04A Rolls/Bagels/Pita/Croutons/Dumplings/ Matzo/Tortilla	36O Juices, Tomato & Vegetable			
04B Crackers/Crispbreads	39A Potato			
04C Muffins/English Muffins	42B Salty/High-Fat Snacks (Incl Tortilla Chips)			
04D Pancakes/Waffles	44A Chocolate Bar			
04E Croissants/Piecrusts/Phyllo Dough	50D Sauces			
04F Dry Mixes (Cakes/Muffins/Pancakes)	(White/Bearnaise/Soya/Tartar/Ketchup)			
07B Biscuits, Commercial	51B Coffee			
08A Pies, Commercial				

Table S13. Comparison of daily protein intake, protein quality, and nutrient intake of Canadians who consumed at least one plant food from a BNS food group that qualified for a "Source of Protein" claim in Canada, "high source of protein" claim in Europe, or each respective claim in both regions

			Plant Protein Content Claim Consumers						P-value
	Non-consumers (n=1,885)[1]		Canada (n=7)		EU (n=8,684)		Canada + EU (n=1,241)		
**Protein Quality**									
	**Mean**	**SE**	**Mean**	**SE**	**Mean**	**SE**	**Mean**	**SE**	
**Total Protein Intake (g/d)**	74.1[b]	4.4	54.3[ab]	11.3	80.1[a]	1.2	84.7[a]	2.5	<0.001
Protein intake from animal foods (g/d)	51.4	3.4	29.9	11.8	51.8	1.3	49.1	2.5	0.202
Protein intake from plant foods (g/d)	21.7[b]	1.0	24.3[abc]	6.4	27.5[c]	0.3	35.0[a]	0.9	<0.001
**Total Protein Intake (% E)**	16.8	0.4	15.1	2.6	17.1	0.2	16.7	0.2	0.641
Protein intake from animal foods (% E)	11.7[a]	0.4	8.3[abc]	3.2	11.2[ac]	0.2	9.7[b]	0.3	0.009
Protein intake from plant foods (% E)	4.98[c]	0.11	7.05[abc]	1.12	6.00[b]	0.05	7.20[a]	0.21	<0.001
**Dietary Protein Quality (DIAAS)[2]**	0.959	0.004	0.887	0.086	0.959	0.002	0.961	0.006	0.791
Protein quality from animal foods (DIAAS)[2]	0.986	0.006	0.988	0.045	0.991	0.002	0.988	0.006	0.784
Protein quality from plant foods (DIAAS)[2]	0.577[c]	0.004	0.593[bc]	0.027	0.598[b]	0.003	0.695[a]	0.007	<0.001
**Corrected Protein Intake (g/d)[3]**	71.2[a]	4.1	50.3[ab]	13.4	76.9[b]	1.3	81.6[b]	2.8	0.001
Protein intake from animal foods (g/d)[3]	51.1	3.2	29.8	11.9	51.6	1.2	48.9	2.5	0.207
Protein intake from plant foods (g/d)[3]	12.4[b]	0.6	14.0[abc]	3.5	16.4[c]	0.2	24.4[a]	0.7	<0.001
**Corrected Protein Intake (% E)**	16.2	0.4	14.1	3.3	16.6	0.2	16.3	0.3	0.516
Protein intake from animal foods (% E)	11.6[a]	0.4	8.2[ab]	3.2	11.1[a]	0.2	9.6[b]	0.3	0.011
Protein intake from plant foods (% E)	2.86[c]	0.05	4.13[ab]	0.56	3.58[b]	0.03	5.07[a]	0.16	<0.001
**Macronutrients**									
Energy (kcal)	1803.6[a]	107.8	1376.8[ab]	221.2	1884.8[b]	16.9	2024.0[b]	52.1	<0.001
Carbohydrates (g)	208.4[a]	6.0	180.5[ab]	28.3	223.6[ab]	2.7	243.6[b]	6.0	<0.001
Carbohydrates (% E)	47.2	1.9	53.3	3.9	47.7	0.5	48.0	1.1	0.302
Total Fibre (g/1000 kcal)	7.6 [ab]	0.6	8.4[ab]	1.6	9.9[b]	0.1	11.5[a]	0.4	<0.001
Total Sugars (% E)	19.3	0.9	23.3	2.5	18.9	0.2	18.1	0.6	0.054
Fat (g)	69.6[a]	7.6	51.4[ab]	13.1	69.8[ab]	0.9	76.7[b]	3.2	0.032
Fat (% E)	33.0	1.8	31.6	4.9	32.0	0.3	32.8	0.9	0.707
Saturated Fat (% E)	10.9	0.3	8.2	1.5	10.3	0.1	10.4	0.4	0.131

Polyunsaturated Fat (% E)	6.9^{ab}	0.8	7.9^{ab}	1.5	6.87^{b}	0.08	7.3^{a}	0.2	0.001
Linoleic Acid (% E)	6.0^{a}	0.8	6.9^{a}	1.4	5.83^{a}	0.08	6.1^{a}	0.2	0.002
Linolenic Acid (% E	1.31^{b}	0.08	1.13^{abc}	0.31	1.60^{c}	0.03	1.91^{a}	0.08	<0.001
Cholesterol (mg/1000 kcal)	160.5^{a}	6.9	117.8^{ab}	52.4	146.4^{ab}	2.5	135.4^{b}	8.1	0.011
Alcohol (%)	3.069^{a}	0.344	0.001^{b}	0.002	3.191^{a}	0.329	2.460^{a}	0.335	0.006
**Micronutrients: per 1000 kcal**									
Vitamin A (ug RAE)	294.4^{b}	14.1	297.3^{ab}	168.0	379.0^{a}	13.2	384.4^{a}	23.9	<0.001
Thiamin (mg)	0.81^{a}	0.04	1.07^{ab}	0.15	0.87^{b}	0.01	0.88^{b}	0.05	<0.001
Riboflavin (mg)	1.03^{a}	0.03	1.24^{a}	0.24	1.07^{a}	0.01	1.03^{a}	0.02	0.043
Niacin (mg)	21.2^{ab}	0.5	20.3^{ab}	2.1	21.7^{a}	0.3	19.8^{b}	0.5	<0.001
Vitamin B-6 (mg)	0.86^{a}	0.03	0.89^{ab}	0.15	0.95^{b}	0.02	0.90^{ab}	0.03	<0.001
Folate (ug DFE)	85.3^{b}	3.9	93.7^{bc}	12.5	124.6^{c}	1.6	143.4^{a}	4.3	<0.001
Vitamin B-12 (mg)	2.25	0.14	1.48	0.59	2.24	0.07	2.09	0.13	0.612
Vitamin C (mg)	38.2^{b}	6.2	36.0^{ab}	22.4	59.0^{a}	1.2	61.3^{a}	2.8	<0.001
Vitamin D (ug)	2.9	0.3	3.0	0.9	2.57	0.09	2.5	0.2	0.676
Calcium (mg)	383.1^{b}	15.8	505.4^{ab}	179.9	428.6^{a}	7.3	476.3^{a}	16.0	<0.001
Iron (mg)	6.30^{a}	0.13	6.38^{abc}	0.90	6.78^{c}	0.05	7.19^{b}	0.12	<0.001
Magnesium (mg)	154.9^{b}	6.4	182.1^{ab}	16.2	174.2^{a}	2.1	185.1^{a}	3.6	<0.001
Phosphorus (mg)	683.1^{a}	11.6	716.9^{ab}	159.9	696.8^{ab}	5.4	712.0^{b}	9.7	0.034
Potassium (mg)	1350.2^{b}	66.6	1688.1^{ab}	197.7	1528.0^{b}	11.3	1591.1^{a}	27.4	<0.001
Zinc (mg)	5.42^{a}	0.16	4.53^{ab}	0.67	5.62^{ab}	0.04	6.02^{b}	0.17	<0.001
Sodium (mg)	1402.0^{a}	34.3	1336.8^{ab}	333.4	1486.2^{b}	12.4	1522.5^{b}	26.5	<0.001
Monounsaturated Fat (% E)	12.4	0.7	12.9	2.7	11.9	0.2	12.1	0.3	0.791

Abbreviations: BNS, Bureau of Nutritional Science; DIAAS, digestible corrected amino acid score; DFE, dietary folate equivalents; EU, European Union; % E, percent energy; IAA, indispensable amino acids; SE, standard error ANCOVA was conducted to compare protein quality and quantity among comparison groups. All models were adjusted for the following variables: misreporting status (EI/TEE), age, sex, smoking, self-perceived health, blood pressure, diabetes, heart disease, cancer, osteoporosis, education, physical activity, income, BMI, immigrant status, and weekend reference day, as previously described [18].Post-hoc analysis with Bonferroni adjustment was used for multiple comparisons. Different superscripts within a row indicate significant differences at p<0.05.

[1] Non-consumers did not consume any food from any plant BNS food groups that qualified for a protein content claim in Canada or Europe.

[2] DIAAS was calculated using a 0.8 true nitrogen digestibility coefficient for all foods. Median IAA for BNS food groups were applied to all foods within BNS food group. Protein and IAA intake across all BNS food groups were summed. The IAA requirements for children (> 3 years), adolescents, and adults were used to determine the amino acid score [17]. The lowest amino acid score was considered the DIAAS. DIAAS values were truncated at 1.0 as per recommendations for mixed diets.

[3] Corrected protein intake was obtained by multiplying the daily DIAAS by total daily protein (g) intake, the animal protein DIAAS by protein intake (g) from animal foods, or plant protein DIAAS by protein intake (g).

References

1. Marinangeli, C.P.F.; Fabek, H.; Ahmed, M.; Sanchez-Hernandez, D.; Foisy, S.; House, J.D. The effect of increasing intakes of plant protein on the protein quality of Canadian diets. *Applied Physiology, Nutrition, and Metabolism* **2021**, *46*, 771-780, doi:10.1139/apnm-2020-1027 %M 33591857.

2. Government of Canada. Food and Drug Regulations. In *Part B, Division 1, Nutrient content claims, B.01.001. Main Dish*, Canada, G.o., Ed. Government of Canada: Ottawa ON, Canada 2024.

3. Government of Canada. Nutrition labelling - Table of reference amounts for food. Availabe online: https://www.canada.ca/en/health-canada/services/technical-documents-labelling-requirements/nutrition-labelling-table-reference-amounts-food.html#c (accessed on October).

4. Government of Canada. Food and Drug Regulations. Schedule K: Reasonable Daily Intake for Various Foods. Availabe online: https://laws.justice.gc.ca/eng/regulations/c.r.c.,_c._870/page-105.html#h-580603 (accessed on February 11, 2025).

5. Canadian Food Inspection Agency. Elements within the Nutrition Facts table: Protein. Availabe online: https://inspection.canada.ca/en/food-labels/labelling/industry/nutrition-labelling/elements-within-nutrition-facts-table#c7 (accessed on February 10, 2025).

6. Canadian Food Inspection Agency. Specific nutrient content claim requirements: Protein claims. Availabe online: https://inspection.canada.ca/en/food-labels/labelling/industry/nutrient-content/specific-requirements#a3 (accessed on July 14, 2025).

7. Canadian Food Inspection Agency. Daily Intake: Reasonable Daily Intake (Schedule K). Availabe online: https://inspection.canada.ca/en/food-labels/labelling/industry/nutrition-labelling/nutrition-facts-table#s14c6 (accessed on February 10, 2025).

8. FAO; WHO. *Protein quality evaluation: Report of the Joint FAO/WHO Expert Consultation, FAO Food and Nutrition: Paper 51 - Table 8*; 0254-4725; Food and Agriculture Organization of the United Nations and The World Health Organization: Rome, Italy, 1991; pp 1-66.

9. FAO; WHO. *Protein quality evaluation: Report of the Joint FAO/WHO Expert Consultation, FAO Food and Nutrition: Paper 51 - Table 9*; 0254-4725; Food and Agriculture Organization of the United Nations and The World Health Organization: Rome, Italy, 1991; pp 1-66.

10. Nosworthy, M.G.; Medina, G.; Franczyk, A.J.; Neufeld, J.; Appah, P.; Utioh, A.; Frohlich, P.; House, J.D. Effect of processing on the in vitro and in vivo protein quality of red and green lentils (Lens culinaris). *Food Chem* **2018**, *240*, 588-593, doi:10.1016/j.foodchem.2017.07.129.

11. Rutherfurd, S.M.; Fanning, A.C.; Miller, B.J.; Moughan, P.J. Protein digestibility-corrected amino acid scores and digestible indispensable amino acid scores differentially describe protein quality in growing male rats. *J Nutr* **2015**, *145*, 372-379, doi:10.3945/jn.114.195438.

12. House, J.D.; Hill, K.; Neufeld, J.; Franczyk, A.; Nosworthy, M.G. Determination of the protein quality of almonds (Prunus dulcis L.) as assessed by in vitro and in vivo methodologies. *Food Sci Nutr* **2019**, *7*, 2932-2938, doi:10.1002/fsn3.1146.

13. Nosworthy, M.G.; Neufeld, J.; Frohlich, P.; Young, G.; Malcolmson, L.; House, J.D. Determination of the protein quality of cooked Canadian pulses. *Food Sci Nutr* **2017**, *5*, 896-903, doi:10.1002/fsn3.473.

14. Nosworthy, M.G.; Medina, G.; Franczyk, A.J.; Neufeld, J.; Appah, P.; Utioh, A.; Frohlich, P.; House, J.D. Effect of Processing on the In Vitro and In Vivo Protein Quality of Beans (Phaseolus vulgaris and Vicia Faba). *Nutrients* **2018**, *10*, doi:10.3390/nu10060671.

15. Nosworthy, M.G.; Franczyk, A.J.; Medina, G.; Neufeld, J.; Appah, P.; Utioh, A.; Frohlich, P.; House, J.D. Effect of Processing on the in Vitro and in Vivo Protein Quality of Yellow and Green Split Peas (Pisum sativum). *J Agric Food Chem* **2017**, *65*, 7790-7796, doi:10.1021/acs.jafc.7b03597.

16. FAO; WHO. *Protein quality evaluation: Report of the Joint FAO/WHO Expert Consultation, FAO Food and Nutrition: Paper 51*; 0254-4725; Food and Agriculture Organization of the United Nations and The World Health Organization: Rome, Italy, 1991; pp 1-66.

17. FAO. *Dietary protein quality evaluation in human nutrition: Paper 92*; Food and Agriculture Organization of the United Nations and The World Health Organization: Rome, Italy, 2013.

18. Wang, Y.F.; Chiavaroli, L.; Roke, K.; DiAngelo, C.; Marsden, S.; Sievenpiper, J. Canadian Adults with Moderate Intakes of Total Sugars have Greater Intakes of Fibre and Key Micronutrients: Results from the Canadian Community Health Survey 2015 Public Use Microdata File. *Nutrients* **2020**, *12*, doi:10.3390/nu12041124.
